# Single-cell analysis of pancreatic ductal adenocarcinoma identifies a novel fibroblast subtype associated with poor prognosis but better immunotherapy response

**DOI:** 10.1038/s41421-021-00271-4

**Published:** 2021-05-25

**Authors:** Yu Wang, Yiyi Liang, Haiyan Xu, Xiao Zhang, Tiebo Mao, Jiujie Cui, Jiayu Yao, Yongchao Wang, Feng Jiao, Xiuying Xiao, Jiong Hu, Qing Xia, Xiaofei Zhang, Xujun Wang, Yongwei Sun, Deliang Fu, Lei Shen, Xiaojiang Xu, Jing Xue, Liwei Wang

**Affiliations:** 1 State Key Laboratory of Oncogenes and Related Genes, Shanghai Cancer Institute, Department of Oncology, Renji Hospital, School of Medicine, Shanghai Jiao Tong University, Shanghai, China; 2 Shanghai Key Laboratory of Pancreatic Disease, Shanghai, China; 3 Department of Bioinformatics and Biostatistics, Shanghai Jiao Tong University, Shanghai, China; 4 Department of Biliary-Pancreatic Surgery, Renji Hospital, School of Medicine, Shanghai Jiao Tong University, Shanghai, China; 5 Department of Pancreatic Surgery, Pancreatic Disease Institute, Huashan Hospital, Shanghai Medical College, Fudan University, Shanghai, China; 6 Shanghai Institute of Immunology, Shanghai Jiao Tong University School of Medicine, Shanghai, China; 7 Zhanjiang Central Hospital, Guangdong Medical University, 2 Cunjin Rd, Chikan District, Zhanjiang, Guangdong Province China; 8 State Key Laboratory of Oncogenes and Related Genes, Stem Cell Research Center, Renji Hospital, School of Medicine, Shanghai Cancer Institute, Shanghai Jiao Tong University, Shanghai, China

**Keywords:** Pancreatic cancer, Cancer immunotherapy

## Abstract

The current pathological and molecular classification of pancreatic ductal adenocarcinoma (PDAC) provides limited guidance for treatment options, especially for immunotherapy. Cancer-associated fibroblasts (CAFs) are major players of desmoplastic stroma in PDAC, modulating tumor progression and therapeutic response. Using single-cell RNA sequencing, we explored the intertumoral heterogeneity among PDAC patients with different degrees of desmoplasia. We found substantial intertumoral heterogeneity in CAFs, ductal cancer cells, and immune cells between the extremely dense and loose types of PDACs (dense-type, high desmoplasia; loose-type, low desmoplasia). Notably, no difference in CAF abundance was detected, but a novel subtype of CAFs with a highly activated metabolic state (meCAFs) was found in loose-type PDAC compared to dense-type PDAC. MeCAFs had highly active glycolysis, whereas the corresponding cancer cells used oxidative phosphorylation as a major metabolic mode rather than glycolysis. We found that the proportion and activity of immune cells were much higher in loose-type PDAC than in dense-type PDAC. Then, the clinical significance of the CAF subtypes was further validated in our PDAC cohort and a public database. PDAC patients with abundant meCAFs had a higher risk of metastasis and a poor prognosis but showed a dramatically better response to immunotherapy (64.71% objective response rate, one complete response). We characterized the intertumoral heterogeneity of cellular components, immune activity, and metabolic status between dense- and loose-type PDACs and identified meCAFs as a novel CAF subtype critical for PDAC progression and the susceptibility to immunotherapy.

## Introduction

Pancreatic ductal adenocarcinoma (PDAC) remains one of the most fatal malignancies, with a 5-year survival rate of ~8%^1^. Eighty percent of PDACs are unresectable at diagnosis, so most patients with PDAC rely on systemic treatment^2,3^. However, the objective response rate (ORR) of first-line PDAC chemotherapy (gemcitabine combined with albumin-bound paclitaxel) is only ~20%^4^. Recently, PD-1/PD-L1 checkpoint blockade has achieved promising results in many types of malignant tumors^5–7^. However, according to the previous reports^6,8,9^, PDAC patients remain unresponsive or poorly responsive to PD-1 antibody, mainly owing to its “immune-cold” feature with very few intratumoral CD8^+^ T cells^10^. A dense extracellular matrix (ECM) as a physical barrier and stromal cells ultimately inhibit both spontaneous and therapeutically induced antitumor immunity, resulting in an “immune-cold” microenvironment in PDAC^10–13^. Some strategies (e.g., a combination with cytotoxic therapy) have been used to increase the efficacy of immune checkpoint blockade in PDAC, and an improved ORR (9%–25%) has been observed in some cohort studies^14–17^. However, acceptable clinical efficacy has not been observed in unselected patients with PDAC^18^. Thus, a challenge for immunotherapy will be to discover rational biomarkers to select susceptible patients for immunotherapy.

Previous studies have shown that PDAC cells can be divided into two major types: classical and basal-like^19–21^. Furthermore, using a large-scale transcriptomic analysis^22^, PDACs were divided into four subtypes: (1) squamous; (2) pancreatic progenitor; (3) immunogenic; and (4) aberrantly differentiated endocrine exocrine. The above-mentioned studies suggest the clinical value of this system; for example, the squamous type is linked to poor prognosis, and the immunogenic subtype has the potential to benefit from immunotherapy. However, preclinical and clinical evidence indicates this correlation is lacking. PDAC is characterized by abundant tumor-associated desmoplasia, which is a key factor affecting its prognosis and treatment^23–28^. In previous studies^29,30^, researchers found high heterogeneity of the tumor stroma among different PDACs, and some PDACs had no obvious desmoplasia^31–33^. Similarly, in a recent study, five subtypes of PDAC were defined including two subtypes of PDAC with low stromal signal^20^. The relationship between prognosis and the extent of desmoplasia in PDAC is controversial. Some researchers reported that poorly differentiated PDAC with low desmoplasia was more aggressive and had a poor prognosis^32^. Some researchers found that stromal abundance (defined as high, medium, and low) was associated with tumor gland typing and believed that PDAC with a low amount of stroma had a better prognosis^34^. The underlying molecular events of stromal heterogeneity are still elusive, and its clinical significance for prognosis and treatments, especially immunotherapy, needs further investigation.

The intratumoral heterogeneity of cancer-associated fibroblasts (CAFs), as the major players in the PDAC stroma, has also been extensively studied^35^. Recently, several studies have explored the heterogeneity of human PDAC at single-cell resolution, revealing the distinct functions of the CAF subtypes in tumor immunity and progression^36–40^. Three major distinct subpopulations of CAFs have been demonstrated in PDAC: (1) the myofibroblastic subset (myCAFs) characterized by smooth muscle actin expression, high transforming growth factor (TGF) signaling and ECM, (2) the inflammatory subset (iCAFs), characterized by high expressions of inflammatory mediators, such as cytokines, chemokines, and complement complex, and (3) the antigen-presenting subset, characterized by the expression of CD74 and MHC class II. Moreover, a promising study showed that a LRRC15^+^ subset of CAFs, defined both in murine and human PDAC tissues, correlated with poor clinical response to PD-L1 blockade^37^. Although the existence of CAF phenotypic heterogeneity in PDAC has been established, the impact of each subset on prognosis and therapeutic response, especially immunotherapy, remains unclear.

Sensitive markers are still lacking for selecting the susceptible PDAC population for immunotherapy. CAFs in PDAC with dense stroma (high desmoplasia) create a physical barrier, which may contribute to the poor response to immunotherapy, whereas PDAC with loose stroma (low desmoplasia) seems to have less of a physical barrier in histopathological analysis and may have a potential opportunity for immunotherapy. However, the responses of PDACs with different degrees of desmoplasia to immunotherapy and the underlying mechanisms are still unclear. In this study, through single-cell RNA sequencing (scRNA-seq), we investigated the intertumoral heterogeneity among patients with different extents of desmoplasia (dense-type, high desmoplasia; loose-type, low desmoplasia), as well as its impact on prognosis and response to therapy. We detected remarkable intertumoral heterogeneity in CAFs, ductal cancer cells, and immune cells between dense- and loose-type PDACs. Notably, no difference in CAF abundance was observed, but a novel subtype of CAFs with a highly activated metabolic state (meCAFs) was found in loose-type PDAC compared with dense-type PDAC. Most importantly, high expression of the meCAF signature was associated with poor prognosis but better response to PD-1 blockade treatment in patients with PDAC.

## Results

### Single-cell analysis uncovers the intertumoral heterogeneity between dense- and loose-type human PDAC

Recent scRNA-seq studies of human PDAC revealed intratumoral heterogeneity in PDAC tumors, which is pivotal for dissecting tumor-related mechanisms in detail^37–41^. The hallmark of PDAC is extensive desmoplasia caused by CAFs; however, we often encounter PDAC tumors with distinct histological features in clinical practice. A cohort study reported that PDACs with low desmoplasia accounted for ~20% of all PDAC tumors^32^. Previously, dense-type and loose-type PDACs were classified according to histological features, but there are no molecular biomarkers to indicate their potential differences in prognosis and therapeutic selection^31–33,42^. To explore the intertumoral heterogeneity with different textures in PDACs, we first divided PDACs into the dense and loose types based on tumor histopathology (Haemotoxylin and Eosin; H&E and Masson stainings).

**Fig. 1:**
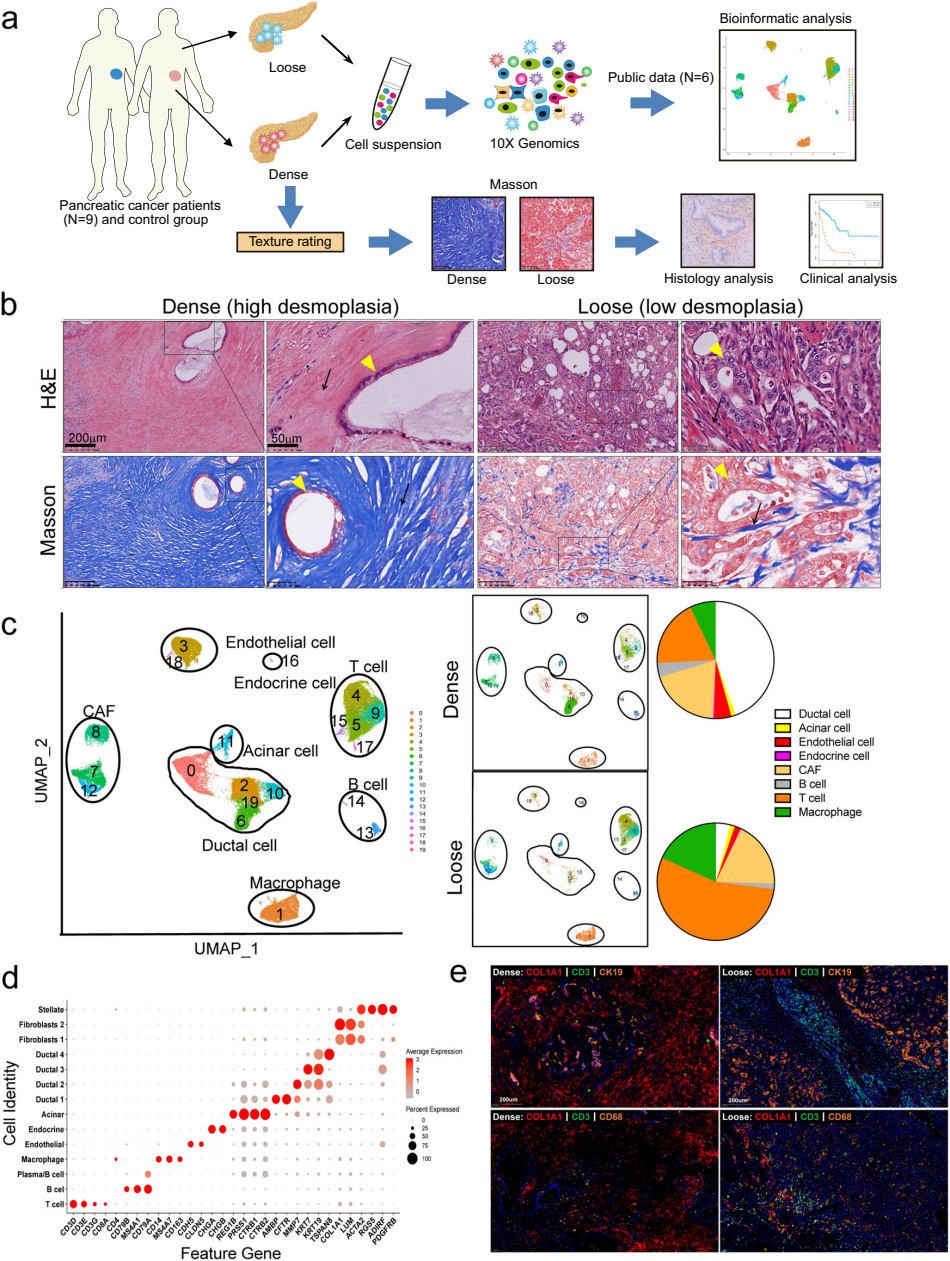
Single-cell analysis reveals cellular heterogeneity between dense- and loose-type human PDACs. **a** Graphical scheme describing the workflow. Nine human PDAC samples with dense or loose stroma, as well as one adjacent normal sample were dissociated into single cells followed by scRNA-seq using the 10× Genomic platform. Public scRNA-seq data from four PDAC and two normal tissues were included for comparison and joint analyses. **b** Representative H&E and Masson staining of four dense- and three loose-type PDAC tumors. Yellow arrow, cancer cell; Black arrow, stroma. **c** Unsupervised clustering of viable cells from nine human PDAC tumors, one adjacent normal sample, and six samples from public data, represented as UMAP plots. Twenty clusters and eight cell types were identified. The cell proportions from dense- and loose-type PDACs are listed. **d** Bubble plot showing selection of cell type-specific markers across major clusters. The size of the dot indicates the fraction of cells expressing a particular marker, and the intensity of the color represents the level of mean expression. **e** Immunofluorescence staining was used to confirm the changes in the proportion of different cell types in dense- and loose-type PDACs. Nine PDAC samples undergoing scRNA-Seq were stained with indicated antibodies (anti-COL1A1 for pan-CAF, anti-CD3 for T cell, anti-CK19 for epithelial cell, anti-CD68 for macrophage). Representative figures are shown. The results show that there are more infiltrating macrophages and T cells in loose-type PDAC than in dense-type.

Nine tumors from untreated PDAC patients (Supplementary Table S1), including four dense-type PDACs, three loose-type PDACs, and two moderate-type PDACs (between dense- and loose-type PDAC) as well as one adjacent normal sample, were enzymatically digested into single-cell suspensions followed by single-cell sequencing using the 10× Genomic platform (Fig. 1a, b). To increase our analytic power, data from all ten samples were combined with public data (four public PDAC samples and two normal pancreatic samples) (Supplementary Table S2). After quality control and removal of low-quality cells, a total of 77,121 cells (our 38,831 cells together with 38,290 cells from public data) were retained for downstream analyses (Supplementary Figs. S1, S2, and Table S3). Batch effects among the samples were observed and corrected. Graph-based clustering of cells identified 20 subclusters with uniform manifold approximation and projection (UMAP) signature genes in each cluster, cross-referenced with known markers of the cell population from the literature, and was used to annotate the different cell types (Fig. 1c; Supplementary Fig. S3 and Table S4). Eight major cell types were characterized: ductal, acinar, endocrine, endothelial, B cell, T cell, fibroblast, and monocyte/macrophage (Fig. 1d; Supplementary Fig. S3 and Table S5). To our surprise, the proportion of CAFs was comparable between the two types of PDACs. However, the proportion of immune cells, including T cells and monocytes/macrophages, was much higher in loose-type PDACs than in dense-type PDACs (Fig. 1c). Then immunofluorescence (IF) staining was used to confirm the changes in the proportion of different cell types in dense- and loose-type PDAC. Nine PDAC samples undergoing scRNA-Seq were multiplex stained. The result showed that loose stroma was associated with increases in infiltrating T cells and macrophages (Fig. 1e; Supplementary Fig. S4). Thus, these results suggest the heterogeneity of stromal cell components in dense- and loose-type human PDAC.

### meCAF is identified in loose-type PDAC

**Fig. 2:**
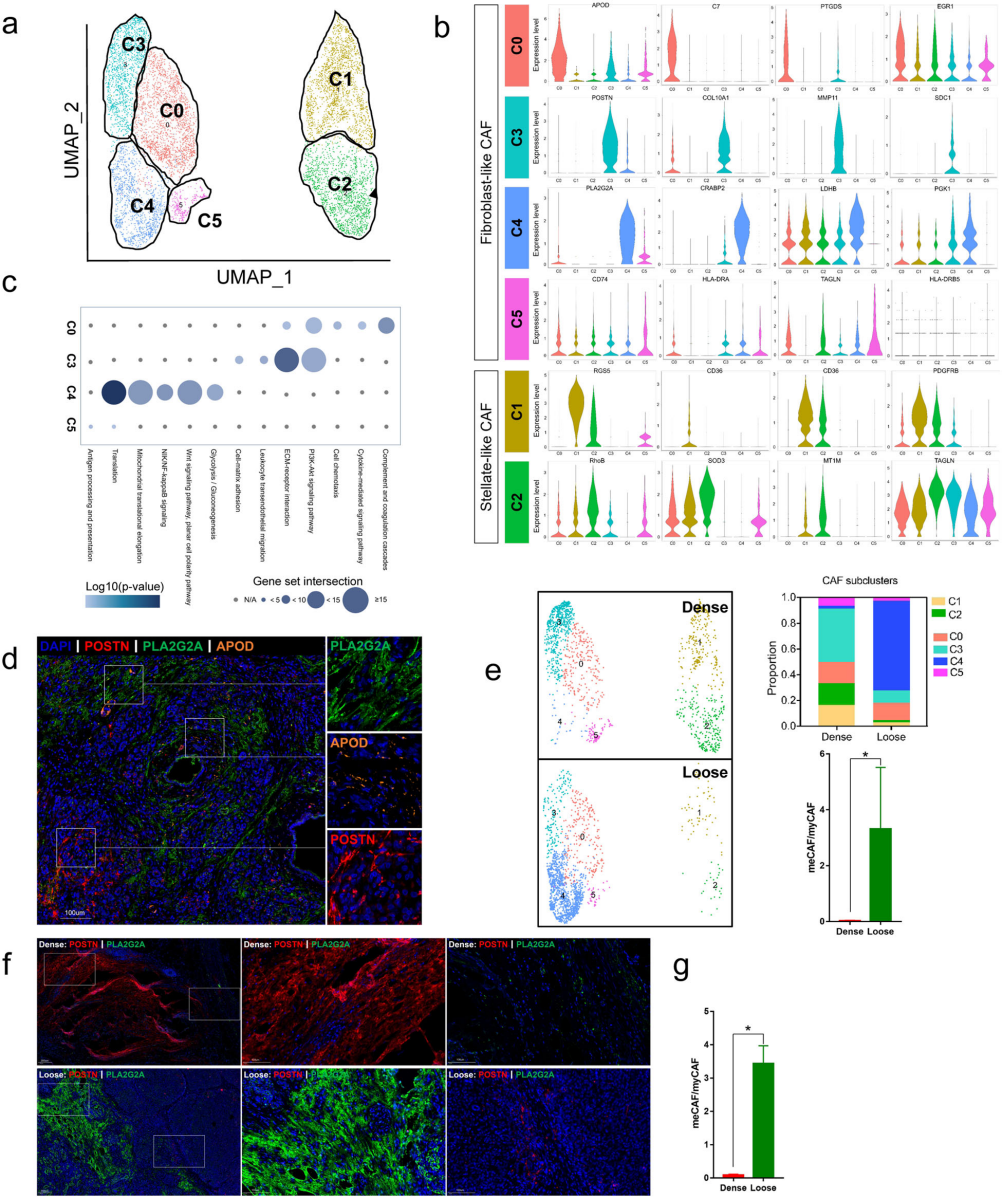
Distinct CAF populations between loose- and dense-type PDACs. **a** Reclustering of CAF cell types in the data set (clusters 8, 7, and 12 from Fig. 1c), represented as a UMAP plot. **b** Violin plot showing normalized expression of marker genes for different CAF subclusters. **c** Hallmark pathways enriched in the four CAF subclusters (subclusters 0, 3, 4, and 5). The size of the dot represents the intersection of marker genes of the subcluster with hallmark pathway gene sets (KEGG and GO), and the intensity of the color indicates log_10_ (*P* value). **d** Nine PDAC samples undergoing scRNA-seq received multiplex immunofluorescence staining to confirm the CAF subgroups in PDACs. Multiplex staining for relevant subCAF markers (APOD for C0 iCAF, orange; POSTN for C3 myCAF, red; PLA2G2A for C4 meCAF, green) showed three distinct populations (the representative image was from one loose-type PDAC). **e** Proportion of six different CAF subclusters in dense- and loose-type PDACs (four dense cases, three loose cases). The ratio of meCAF (C4) to myCAF (C3) for four dense cases was compared with three loose cases. The *y* axis represents the ratio of meCAF/myCAF for *n* = 7 cases (four dense cases and three loose cases). Data are presented as means ± SEM. The result showed that myCAF is the major CAF subgroup in dense-type PDAC while meCAF is the major CAF subgroup in loose-type PDAC. **f** Multiplex immunofluorescence staining was conducted in nine PDAC samples undergoing scRNA-seq to confirm the changes in the CAF subgroups in dense- and loose-type PDACs (POSTN for C3 myCAF, red; PLA2G2A for C4 meCAF, green). Representative images (one dense-type PDAC and one loose-type PDAC from nine PDAC samples) were shown (images of all nine patients were shown in Supplementary Fig. S6), and the results showed that the expression of myCAF marker in dense-type PDAC was high while meCAF marker was highly expressed in loose-type PDAC. **g** The ratio of meCAF (PLA2G2A^+^ CAFs) to myCAF (POSTN^+^ CAFs) for seven cases (four dense-type and three loose-type cases). The *y* axis represents the ratio of meCAF/myCAF in visual fields (40×). Data are presented as means ± SEM. All statistical analyses were performed with the two-sided Mann–Whitney *U* test. *P < 0.05.

To characterize the CAF subpopulations in PDAC, we performed unsupervised clustering analysis and showed that CAFs, which were categorized into stellate-like and fibroblast-like CAFs, could be further divided into six subclusters based on their marker genes. Stellate-like CAFs were divided into two subclusters, C1 (by marker genes *RGS5* and *CD36*) and C2 (by marker genes *MT1M* and *TAGLN*), whereas fibroblast-like CAFs were divided into four subclusters (C0, C3, C4, and C5) (Fig. 2a, b; Supplementary Table S6). In accordance with previous studies^28,40^, we showed that subcluster C3 was myCAFs, which highly expressed *COL10A1* and *POSTN*, and its marker genes were linked to ECM-receptor interaction and PI3K/AKT signaling (Fig. 2a, c). Subcluster C0 was similar to iCAFs, as marker genes in C0 were involved in inflammatory pathways such as complement cascades, cytokine-mediated signaling, and cell chemotaxis pathways (Fig. 2c). Subcluster C5 did not have typical marker genes, and the expression of *CD74* and *HLA-DRA* was slightly increased, indicating its potential weak antigen-presenting function. Most interestingly, we identified a novel subcluster, C4, characterized by high expression of *PLA2G2A* and *CRABP2*, and its marker genes were related to translation, mitochondrial translational elongation and glycolysis, thus C4 was named meCAFs (Fig. 2a, c; Supplementary Tables S7, S8). In order to further confirm the existence of meCAF, myCAF, and iCAF in PDAC, we performed multiplex IF staining with markers for these three subpopulations of CAFs (APOD for C0 iCAF, orange; POSTN for C3 myCAF, red; PLA2G2A for C4 meCAF, green) on nine PDAC samples undergoing scRNA-seq. As shown in Fig. 2d, very few overlapped signals were observed on PDAC samples, indicating three distinct CAF subpopulations in tumor microenvironment (TME) of PDAC (Fig. 2d). Pancreatic stellate cells (PSCs) are thought to be the major precursor of CAFs in PDAC^43^. Consistent with previous findings^35^, a pseudotime analysis of CAFs revealed that stellate cells (C1 and C2) were common precursors of C0, C3, C4, and C5, whereas C0 and C4 were terminally differentiated cell populations (Supplementary Fig. S5). Previous studies had shown that CAFs with high plasticity had the potential to transition from one state to another^41,44^. Here, our findings suggest that C3 has the potential to be converted into C0 or C4, which needs further investigation.

Notably, the C4 subcluster was a dominant CAF population in loose-type PDAC, whereas C3 was the dominant subcluster in dense-type PDAC (*P* = 0.032) (Fig. 2e; Supplementary Table S8). To further confirm our findings, we performed IF staining on all nine samples, which have been used for our scRNA-seq. The representative images from dense- and loose-type PDAC were shown in Fig. 2f and Figure S6 (Fig. 2f; Supplementary Fig. S6). The IF results showed that POSTN was highly expressed in the stroma of dense-type PDAC, whereas PLA2G2A was highly expressed in the stroma of loose-type PDAC, which is consistent with our scRNA-seq analyses (*P* = 0.034) (Fig. 2g). In addition, we found that C4 had a higher proliferative capacity, which might partially contribute to its increased proportion (Supplementary Fig. S5). Furthermore, using gene set enrichment analysis (GSEA), we identified distinct pathways enriched in C3 (myCAF) and C4 (meCAF). AVB3 integrin, focal adhesion, ECM-receptor interaction, PTK2, FAK, PDGF, and MET signaling pathways were significantly enriched in myCAFs, while translation, mitochondrial translation, glycolysis, noncanonical NF-κB, P21/P27 degradation, and MYC pathways were enriched in meCAFs (Supplementary Fig. S5 and Table S9). The demand for increased translational activity in meCAFs may come from their high transcriptional activity (significantly higher unique molecular identifier counts in meCAFs) (Supplementary Fig. S5b). To validate the metabolic feature of meCAF, we co-stained the meCAF marker and myCAF marker with the two main glycolytic genes (LDHA, PKM2), respectively. Compared to myCAF (POSTN^+^ CAFs), the meCAFs (PLA2G2A^+^ CAFs) had more co-staining with LDHA and PKM2, indicating their metabolic feature (Supplementary Fig. S5d). To further investigate the signals driving the difference between the myCAFs and meCAFs, we performed a single-cell regulatory network inference and clustering assay to identify dominant transcription factors (TFs). The analysis revealed that multiple TFs, including FOS, JUN, SOX4, LEF1, and TCF4, were enriched in myCAFs, while a unique enrichment of CREB3L1 was observed in the meCAF population (Supplementary Fig. S5e).

### The cross-talk between tumor cells and CAFs determines the distinct microenvironment between dense- and loose-type PDAC

**Fig. 3:**
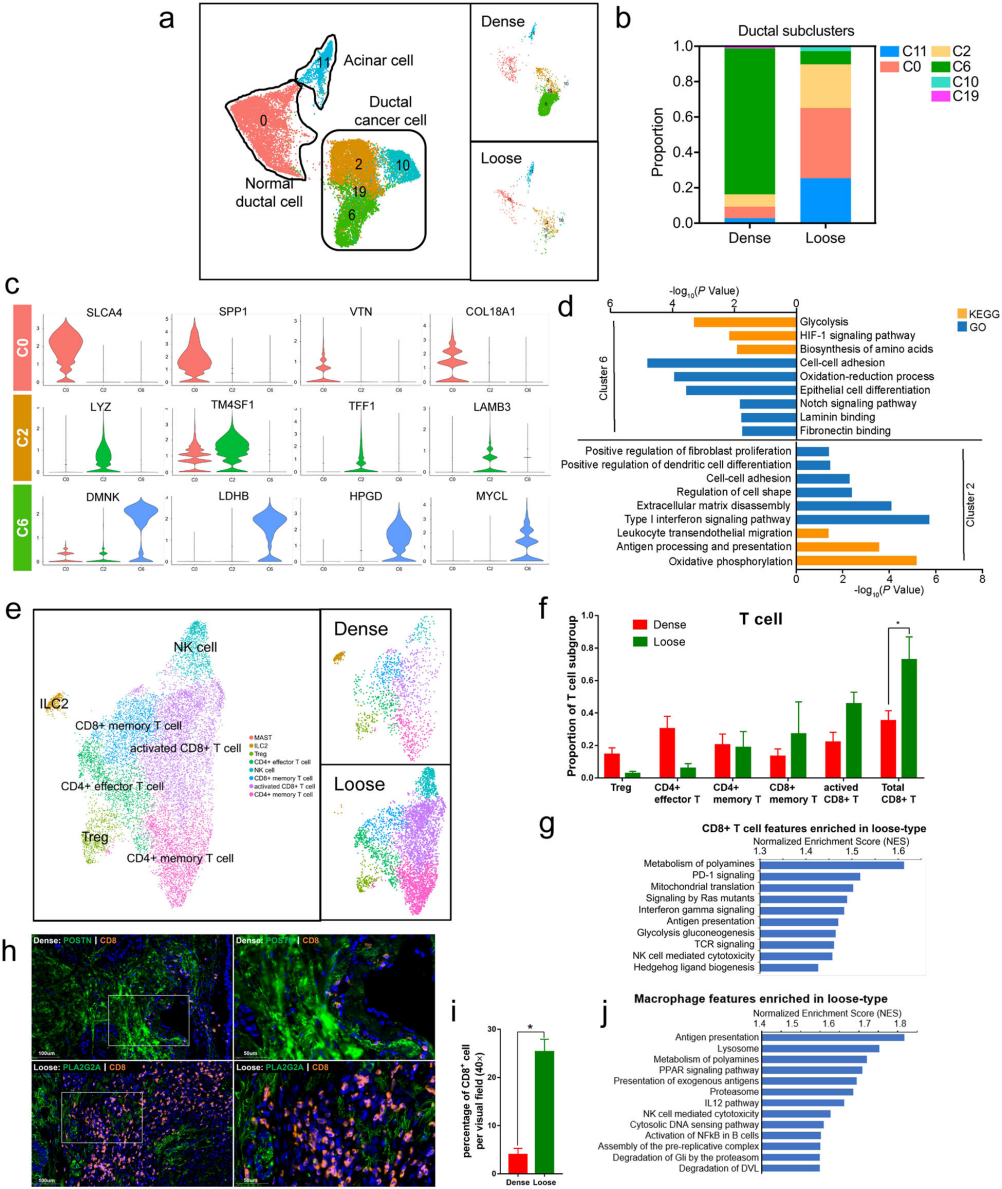
Distinct features of ductal cells and immune cells in dense- and loose-type PDACs. **a** UMAP plot of acinar cells and ductal cells in dense- and loose-type PDACs. **b** Proportion of cells from acinar and ductal cells in dense- and loose-type PDACs. **c** Violin plots of selected marker genes from clusters 0, 2, and 6, showing normalized expression in the different clusters. **d** KEGG and GO analysis comparing cluster 6 against cluster 2. **e** Reclustering of T-cell types in the data set (clusters 4, 5, and 19 from Fig. 1c), represented as a UMAP plot. **f** Proportion of T-cell subpopulations in dense- and loose-type PDACs (four dense cases, three loose cases). The proportion of the CD8^+^ T-cell group in all T cells is significantly increased in loose-type PDAC. The *y* axis represents the proportion of each T-cell subgroup in all T cells, and the *x* axis represents different T-cell subgroups. Red column and green column represent dense-type PDAC and loose-type PDAC, respectively. Data are presented as means ± SEM. All statistical analyses were performed with the two-sided Mann–Whitney *U* test. *P < 0.05. **g** GSEA showing enriched pathways in CD8^+^ T cells comparing loose-type against dense-type PDAC. **h** Multiplex immunofluorescence staining was conducted in nine PDAC samples undergoing scRNA-seq to confirm the correlation between CAF subpopulations and infiltrating CD8^+^ T cells (*n* = 9, POSTN for myCAF, PLA2G2A for meCAF, CD8 for CD8^+^ T cell). Representative immunofluorescence results showed much more infiltrating CD8^+^ T cells in loose-type PDAC than in dense-type PDAC. **i** Quantification of CD8^+^ T cells for *n* = 7 cases (four dense cases and three loose cases), including representative case. The *y* axis represents percentage of CD8^+^ T cell per visual field (40×), and the *x* axis indicates dense-type PDAC and loose-type PDAC, respectively. Data are presented as means ± SEM. All statistical analyses were performed with the two-sided Mann–Whitney *U* test. *P < 0.05. **j** GSEA showing enriched pathways in macrophages comparing loose-type against dense-type PDAC.

CAF heterogeneity is spatially regulated by signals derived from diverse tumor cells. A previous study identified tumor cell-intrinsic TGFβ and IL-1/JAK/STAT3 signaling as the major pathways responsible for myCAF and iCAF formation^40,41^. To elucidate the formation of meCAFs, we uncovered the intertumoral heterogeneity of ductal cancer cells between dense- and loose-type PDACs. According to the classic- and basal-like PDACs previously defined via bulk RNA-seq^45^, two independent scRNA-seq studies revealed that most PDACs in their cohorts harbored classic-like gene signatures^38,40^. In Elyada’s scRNA-seq analysis, ductal cancer cells in human PDAC were clustered in two major subtypes: the classic (by *TFF1*, *TFF2*, *LYZ*, *VSIG2,* and *CEACAM6* marker genes) and secretory (by *SPP1*, *CLU*, *CTGF*, and *COL18A1* marker genes) subtypes. In our study, ductal cancer cells were clustered into four major populations (C2, C6, C10, C19) (Fig. 3a; Supplementary Table S10). By comparing two types of PDAC tumors, we found that C6 subpopulation was dominant in dense-type PDACs, whereas C2 subpopulation was dominant in loose-type PDACs (Fig. 3b). Notably, a subpopulation of C2 expressed classic genes such as *TFF1*, *LYZ*, *TMASF1*, and *LAMB3*, whereas a subpopulation of C6 expressed genes such as *DMNK*, *LDHB*, *HPGD*, and *MYLC*. GO and KEGG analysis of marker genes suggested that highly expressed genes in the C6 subpopulation of ductal cancer cells were involved in glycolysis, biosynthesis of amino acids, HIF-1α signaling, ECM binding, and epithelial cell differentiation. In contrast, the C2 subpopulation was shown to express genes enriched in oxidative phosphorylation, ECM disassembly, type I interferon signaling, antigen presentation, and leukocyte transendothelial migration (Fig. 3c, d; Supplementary Fig S7 and Table S11). These data revealed the unique metabolic characteristics of the C2 and C6 subclusters, which may be related to their distinct location and surrounding TME in tumors. In addition, high expression of ECM disassembly-related genes in the C2 subpopulation might be the reason for the lower desmoplasia in loose-type PDAC. In contrast, elevated gene expression related to ECM–cell interactions in the C6 subpopulation might be critical for the survival of dense-type PDAC cells. Moreover, we found that genes expressed in C2 subpopulations had inflammatory features, including those involved in promoting immune cell trafficking and antigen presentation as well as activating interferon signaling. This result is consistent with our finding that the proportion of T-cell and monocyte/macrophage populations was markedly increased in loose-type PDAC (Fig. 1e, Supplementary Fig. S4).

To elucidate the T-cell heterogeneity between dense- and loose-type PDACs, we performed unsupervised dimensionality reduction and clustering for pan–T-cell populations. T cells can be regrouped into eight distinct subclusters in PDAC (Fig. 3e). The proportion of each subgroup of T cells in dense- and loose-type PDAC was shown in Fig. 3f. Of note, the proportion of the CD8^+^ T-cell group in all T cells was significantly increased in loose-type PDAC (*P* = 0.034) (Fig. 3f). Then the result was further confirmed by IF staining. Multiplex IF staining was conducted in nine PDAC samples undergoing scRNA-seq to confirm the correlation between CAF subpopulations and infiltrating CD8^+^ T cells. The results showed much more infiltrating CD8^+^ T cells in loose-type PDAC than in dense-type PDAC (*P* = 0.034) (Fig. 3h, i; Supplementary Fig. S8 and Tables S12, 13). Moreover, GSEA results showed that metabolism of polyamines, PD-1 signaling, mitochondrial translation, IFNγ signaling, and antigen-processing pathways were enhanced in CD8^+^-activated T cells in loose-type PDAC compared to dense-type PDAC (Fig. 3g; Supplementary Fig. S9 and Table S14). These data indicate that the CD8^+^ T cells in loose-type PDAC not only showed increased proportion but also have an enhanced cytotoxic ability. Similar to the results of CD8^+^ T cells, we also observed that the terms including antigen presentation, lysosomes, metabolism of polyamines, PPAR signaling, and NF-κB signaling were significantly enriched in monocytes/macrophages from loose-type PDAC, indicating their inflammatory characteristics (Fig. 3j; Supplementary Fig. S9 and Table S15).

We next used TCGA database to explore the correlation between four CAF subpopulations (C0, C3, C4, C5) or tumor cell subpopulations (C2, C6) and immune cells, based on their unique marker genes. As shown, only the C0 and C4 subclusters of CAFs had a strong positive correlation with immune cells, whereas the C2 and C6 subclusters of tumor cells showed a lower correlation (Supplementary Fig. S9d). Then, CellChat analysis^46^ was used to explore cell type interactions between T cells and CAFs or ductal cell subtypes. We identified meCAF as the dominant cell type to communicate with T cells, and 80 and 87 ligand–receptor (L–R) pairs were involved in the signaling from meCAFs to T cells and the signaling from T cells to meCAFs, respectively. Moreover, we found that the L–R pairs between ductal cells and T cells were significantly lower than CAFs and T cells. Similar results were identified in macrophages, as meCAFs (L–R; 110 and 131) had more cross-talk with macrophages compared to myCAFs (L–R; 83 and 95) and iCAFs (L–R; 83 and 92). These data indicate that the difference in the proportion of immune cells among PDAC tumors is mainly driven by different subtypes of CAFs rather than tumor cells (Supplementary Fig. S10).

### Loose-type PDACs with meCAFs show a distinct prognosis

Owing to the intertumoral heterogeneity in dense- and loose-type PDACs we defined above, we hypothesized that the difference might affect patients’ prognosis and response to treatments. Dense-type PDAC has the ability to adapt to the TME, which is characterized by hypoxia and poor blood supply, and thus, this subtype might be less sensitive to chemotherapy than other types^47^. Matrix-rich TME reduces the metastatic/invasive ability and decreases access of immune cells to tumor cells;^27^ therefore, this type could be considered as a relatively “immune-cold” tumor. In contrast, with less matrix restriction, loose-type PDACs have a relatively higher ability to invade and metastasize and have more infiltrating immune cells than dense-type PDACs; thus, this type is considered to be an “immune-hot” tumor^10^.

**Fig. 4:**
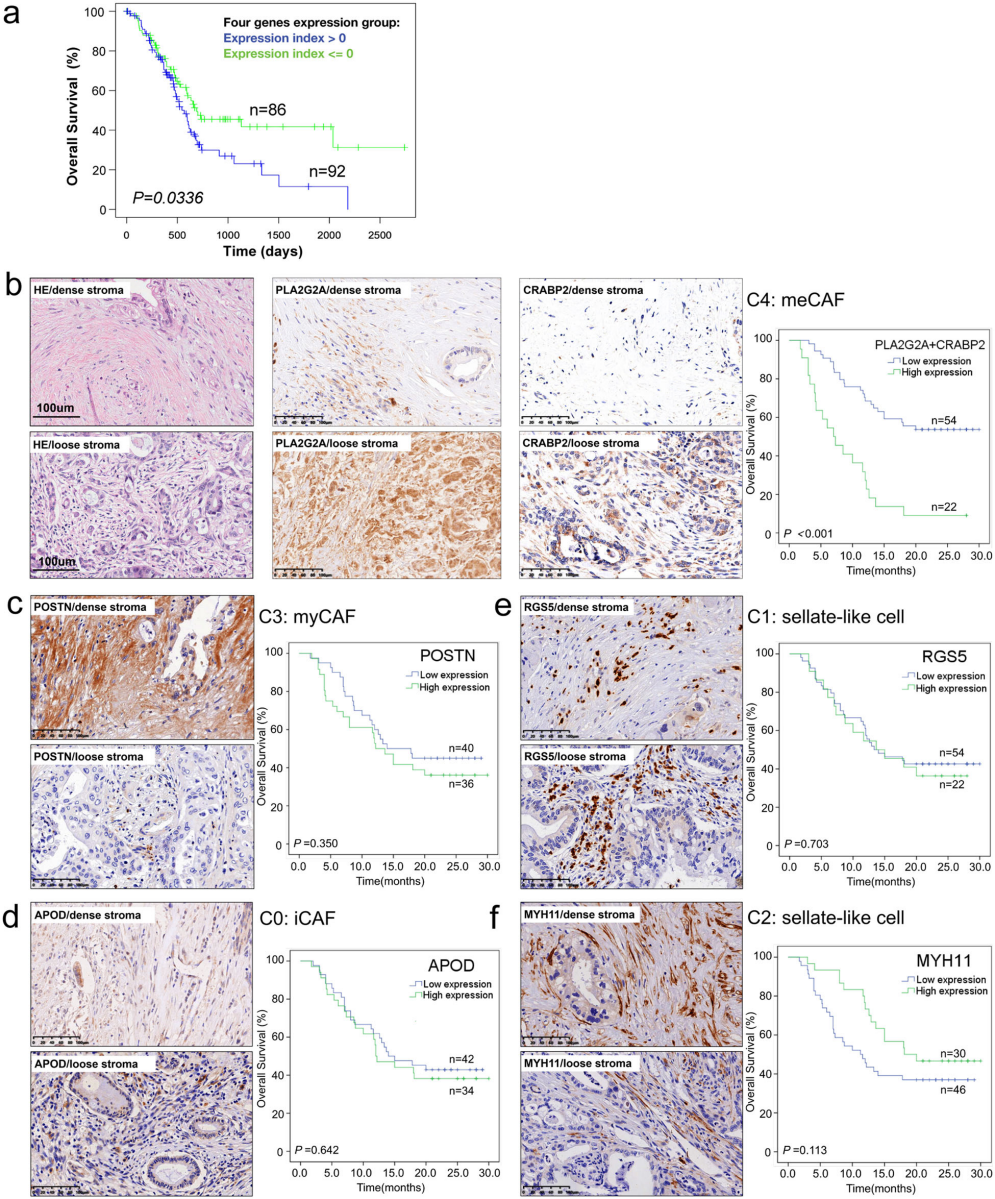
High expression of meCAF markers in the stroma predicts poor prognosis in patients with PDAC. **a** Higher expression of four meCAF marker genes (*PLA2G2A, CRABP2, SERPINE2,* and *MFAP5*) predicting a worse overall survival (OS) in patients with PDAC (TCGA PAAD database, *n* = 178). **b** Representative H&E and IHC stainings of meCAF markers (PLA2G2A and CRABP2) indicated high expression of these markers in loose-type PDAC (*n* = 94). High expression of meCAF markers (PLA2G2A + CRABP2) in the stroma predicted a poor OS in patients with PDAC (*n* = 76). **c** Representative IHC staining of myCAF marker (POSTN) indicated its high expression in dense stroma of PDAC (*n* = 94). The expression level of POSTN in stroma showed no correlation with OS of PDAC patients (*n* = 76). **d**–**f** Representative IHC staining of an iCAF marker (APOD) and CAF subcluster 1 and 2 markers (RGS5 and MYH11). The expression of these markers show no correlation with the overall survival in patients with PDACs. Scale bar, 100 μm. Censored samples are indicated by “+”. Kaplan–Meier curve was used for survival analysis. All statistical analyses were performed with the Log-rank test.

To test our hypothesis, we first followed seven patients (three patients with loose-type PDACs and four patients with dense-type PDACs) who were recruited for our scRNA-seq study. Consistent with our hypothesis, all three patients with loose-type PDAC relapsed after surgery within 6 months (Supplementary Table S1). In addition, we used TCGA database to analyze the association of marker genes in multiple CAF subgroups with patient prognosis. We found that combined marker genes of meCAFs were negatively correlated with patient survival (Fig. 4a). Furthermore, to verify the expression of the marker genes of the above six CAF subgroups, we performed immunohistochemical staining of markers for each subcluster (PLA2G2A and CRABP2 for C4 meCAFs, POSTN for C3 myCAFs, APOD for C0 iCAFs, RGS5 for C1, MYH11 for C2) on our PDAC tissue array (Renji cohort, *n* = 94). We observed that PLA2G2A- and CRABP2-positive fibroblasts were mainly present in PDAC with loose stroma (*P* < 0.001). In contrast, POSTN-positive fibroblasts were abundant in the dense stroma (*P* = 0.012) (Supplementary Table S16). The results of prognostic analysis showed that high expression of the meCAF markers (PLA2G2A + CRABP2) in the stroma was linked with poor overall survival (HR: 3.900, 95% CI: 2.134–7.127) (Fig. 4b). Correlation analysis with clinicopathological characteristics showed that meCAFs were positively correlated with vascular invasion in patients (Supplementary Table S17). However, the other four CAF subgroups were not found to be correlated with overall survival (Fig. 4c–f). These data further indicate that meCAFs might be linked to the higher metastasis potential of PDAC, thereby affecting patient prognosis.

### High abundance of meCAFs is correlated with a better response to immunotherapy in PDAC patients

In clinical practice, PDACs with moderate desmoplasia, which harbor partial characteristics of stroma from dense- and loose-type PDACs, are often encountered (Supplementary Fig. S11). It is difficult to classify these cases into either dense- or loose-type PDACs. Therefore, compared with the degree of desmoplasia, the meCAF signal can be precisely quantified and possibly used to predict the prognosis of PDAC patients and their response to immunotherapy.

**Fig. 5:**
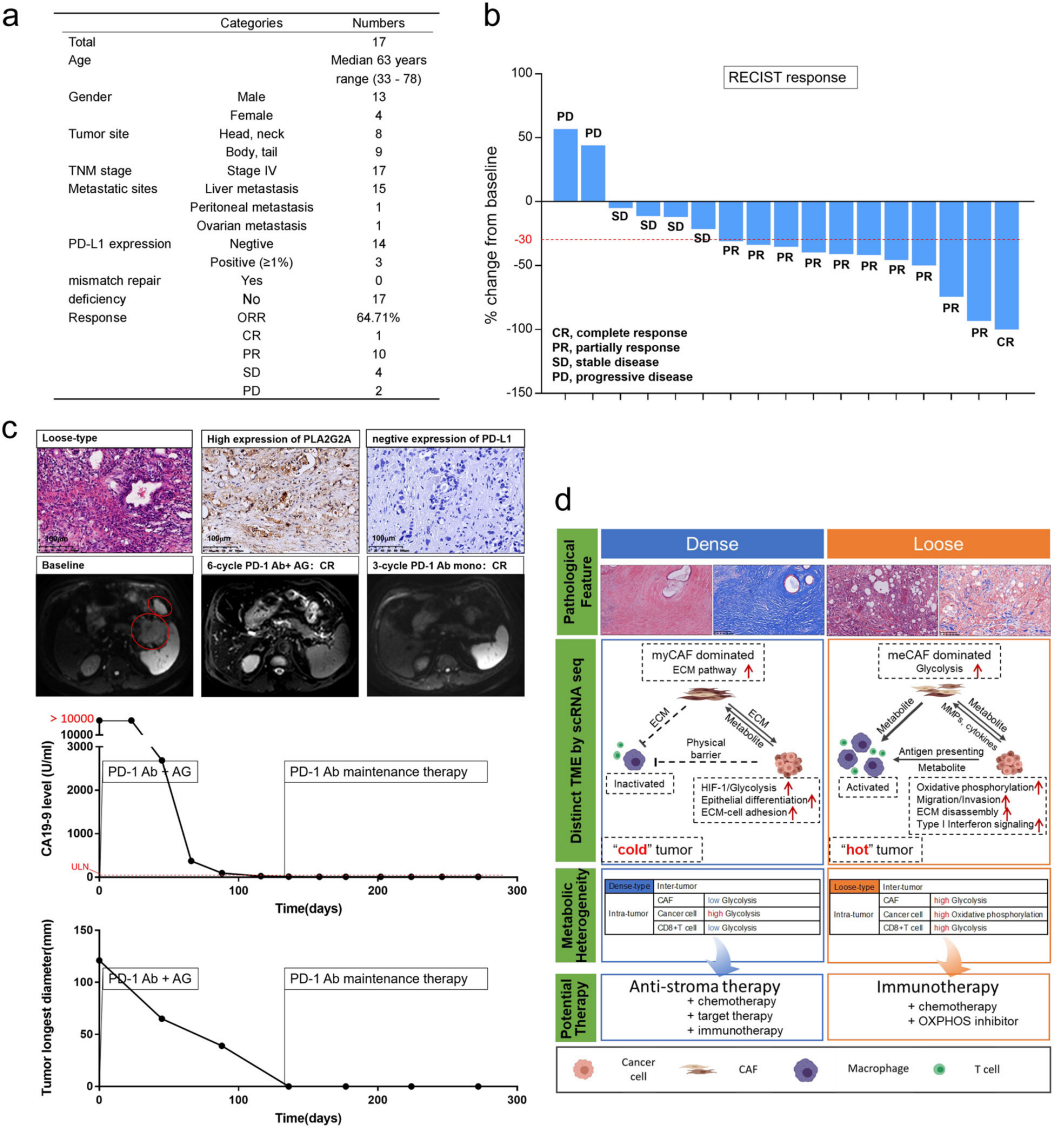
High abundance of meCAFs is correlated with a better response to immunotherapy in patients with PDAC. **a** Clinical characteristic of 17 PDAC patients with abundant meCAFs included in our study. **b** Response evaluation of the 17 PDAC patients receiving 6 cycles of chemotherapy (gemcitabine plus albumin-bound paclitaxel) combined with PD-1 antibody (10 partial responses, 1 complete response, objective response rate: 64.71%). **c** The response of a representative loose-type PDAC patient after receiving chemotherapy combined with PD-1 antibody treatment and PD-1 antibody alone. H&E and IHC stainings of PLA2G2A and PD-L1 were examined. Follow-up clinical indicators including MRI and serum CA-199 after treatment are shown. CR, complete response; AG, gemcitabine plus albumin-bound paclitaxel; ULN, upper limit of normal. **d** Schematic diagram summarizing the intertumoral heterogeneity of cellular components, function and metabolism in dense- and loose-type PDACs, suggesting potential clinical significance and therapeutic strategies.

To explore whether meCAF abundance would affect the response to immunotherapy, we conducted a pilot study to observe patient responses to the PD-1 antibody. Patients aged ≥18 years who had a definite pathologic diagnosis of metastatic PDAC by the use of percutaneous biopsy were eligible. Patients with exposure to prior systemic therapy (including adjuvant therapy) were excluded. H&E staining was first used to distinguish tumor stroma, and then IF staining was used to detect PLA2G2A expression. PD-L1 expression and MMR status were also evaluated. Seventeen PDAC patients with abundant meCAFs (high PLA2G2A expression in the stroma) were selected, and their responses to the PD-1 antibody were evaluated. Detailed clinical information was shown in Fig. 5a. These patients first received six cycles of chemotherapy (gemcitabine plus albumin-bound paclitaxel) combined with PD-1 antibody and then received PD-1 antibody alone as maintenance therapy. To our surprise, a dramatic response was observed in these 17 patients (10 with partial response, one with complete response (CR), ORR: 64.71%), which was much higher than that in previous reports^14,15^ (Fig. 5b, Supplementary Fig. S12). Notably, the patient who achieved a CR was treated with PD-1 antibody as maintenance therapy for > 3 months, and the evaluation was still CR (Fig. 5c). We also evaluated the predictive power of MMR status and PD-L1 expression. However, no correlation was found between these biomarkers and the response to PD-1 antibody (*P* = 0.236) (Supplementary Table S18). These data encourage us to perform a large-scale clinical study to further validate the clinical significance of our findings in the future.

## Discussion

Stromal desmoplasia has been shown to be related to the prognosis of PDAC patients in many studies, but the results remain controversial. To understand the difference in cellular and molecular events in PDACs with different extents of desmoplasia, extremely loose- and dense-type PDACs were selected for single-cell transcriptome analysis. We identified the existence of a novel CAF subtype, meCAF, in loose-type PDACs. We observed the distinct compositions of CAF subtypes in different types of PDACs, in which myCAFs producing a large amount of ECM proteins were the dominant subtype in dense-type PDAC, whereas meCAFs with high metabolic activity were the major subtype in loose-type PDAC. The abundance of meCAFs was linked to poor clinical outcomes in PDAC patients but was associated with better immunotherapy responses in our pilot study. Our findings improve our understanding of PDAC intertumoral heterogeneity and provide a potential predictive biomarker for immunotherapy in patients with PDAC.

The cross-talk among different types of cells in the TME affects tumor progression^48,49^. Our scRNA-seq comparison analyses revealed different patterns of cross-talk among cells in the TME, resulting in distinct microenvironments between dense- and loose-type PDACs. Mutations in cancer cells lead to tumor arising; therefore, cancer cells are considered the direct source of cues to reprogram other stromal cells^34,41^. As expected, our study found that the intertumoral heterogeneity of cancer cells might be one of the major determining factors for CAF diversity between the two types of PDACs. We performed targeted next-generation sequencing (NGS) but did not observe any relationship between genetic mutations and these two types of PDACs (Supplementary Table S1). The result raised a hypothesis that loose-type PDACs may have differences in epigenetic modifications rather than genomic alterations, compared with dense-type PDAC. Meanwhile, some studies have shown that stromal desmoplasia can also affect the subtypes of tumor cells^29^. Thus, the causal relationship between CAFs and cancer cells needs further investigation. Furthermore, we found that meCAFs instead of tumor cells played a dominant role in forming a relatively “hot” immune microenvironment in loose-type PDAC. Recent studies have shown that cancer cells and CAFs are metabolically coupled. CAFs, through aerobic glycolysis, generate high levels of energy-rich fuels for mitochondrial OXPHOS in adjacent cancer cells^50–52^. Thus, in loose-type PDACs, abundant meCAFs might produce a large amount of catabolic fuel to support mitochondrial OXPHOS activity in cancer cells. In dense-type PDAC, cancer cells closely surrounded by CAFs under hypoxic conditions mainly rely on HIF-1a-dependent aerobic glycolysis to produce energy for cellular processes^28,44^. As aerobic glycolysis requires more carbolic fuel than OXPHOS to produce the same amount of ATP^53,54^, we speculate that the increased glycolytic ability of cancer cells weakens the metabolic capacity of other stromal cells via nutrient competition. Our findings indicate that myCAFs and meCAFs promote the development of PDAC in two distinct ways: myCAFs promote the growth of PDAC by producing ECM via a juxtracrine mechanism and blocking immune cell infiltration via a physical barrier, whereas meCAFs produce metabolic intermediates as a fuel source for cancer cells and immune cells to favor PDAC progression (Fig. 5d).

PD-1/PD-L1 checkpoint blockade has achieved promising results in many types of malignant tumors. However, for PDAC, immunotherapy faces the dilemma of poor response and unknown effective population. The PD-L1 protein level is widely used as a biomarker of PD-1 checkpoint blockade therapy^55,56^. However, the expression of PD-L1 does not exist in most PDACs^57^. In our pilot study, only three patients with PD-L1-positive cancer cells >1% were detected. Mismatch repair (MMR) status can be used to predict the efficacy of PD-1 checkpoint immunotherapy^58^, but <1% of PDAC patients harbor MMR deficiency. In our study, the abundance of meCAF accounted for a relatively “hot” immune microenvironment. Thus, we enrolled PDAC patients with positive expression of stromal PLA2G2A (a meCAF marker) for PD-1 checkpoint blockade plus chemotherapy treatment and observed a greatly improved response to immunotherapy (ORR: 64.71%). This promising result provides a feasible strategy for using stromal markers to screen the target population for immunotherapy (Supplementary Fig. S13). Moreover, our study showed that cancer cells in PDAC with abundant meCAFs used OXPHOS rather than glycolysis, suggesting that these PDAC cells might be more sensitive to OXPHOS inhibitors such as metformin. Therefore, PDAC patients with abundant meCAFs might benefit from a combination of immune checkpoint blockade and OXPHOS inhibitors. We will further conduct a large randomized controlled study to confirm our hypothesis.

This study has some limitations: (1) We defined the meCAF subset, described its feature and potential communications with immune cells based on our scRNA-seq data. However, more direct evidence is needed to further confirm the metabolic characteristics of meCAF and its cross-talk with immune cells and tumor cells. (2) Our preliminary study aims to explore the potential of stromal PLA2G2A expression as a companion biomarker for immunotherapy. Even though it is a single-arm, single-center, non-randomized pilot study with limited power, the promising result of the trial encourages us to further carry out randomized controlled trials with more-enrolled patients in the future.

Overall, our findings reveal intertumoral heterogeneity among PDAC patients with different extents of desmoplasia and define a novel subtype of meCAFs. The abundance of meCAFs was associated with poor overall survival in patients with PDAC but predicted a better response to immunotherapy for patients. Future studies focusing on meCAF function and regulatory mechanisms are sure to yield a more complete understanding of interactions between meCAFs and other cell types and contribution to the prediction of tumor progression and response to therapy.

## Materials and methods

### Human samples and tissue texture rating

Human PDAC resection samples were obtained from Renji and Huashan Hospitals under informed consent from all patients. All tissue experiments were reviewed and approved by the Ethics Committee of Renji Hospital. The clinical characteristics of individual patients are shown in Supplementary Table S1. Samples were confirmed based on pathologist assessment, and tissue texture was preliminarily rated as 1 (loose), 2 (moderate), and 3 (dense) by three independent reviewers. In brief, fresh specimens of PDAC and control pancreases were collected at the time of surgical resection. The looseness of the cut surface texture and the ease of cutting were used as scoring criteria. Then, three experienced pathologists evaluated the matrix density based on H&E and Masson trichrome staining and classified the samples as loose-, moderate-, or dense-type^59^. PDAC with loose stroma exhibited low desmoplasia and a high level of cellular components. The morphological appearance of loose stroma is characterized by a loose fibroblastic myxoid stroma and occasional short wispy collagen fibers. PDACs with dense stroma showed a densely packed network of fibers with intense staining. Thus, dense stroma presents mature collagen fibers packed into multilayers with intense staining, and others were considered to be “moderate-type”.

### Masson’s trichrome staining

Formalin-fixed tissue was embedded in paraffin, and 5-μm sections were stained with Masson’s trichrome reagent to visualize collagen. In brief, sample sections were deparaffinized and rehydrated, refixed in Bouin’s liquor overnight, and washed in running water to remove the yellow color. The slides were stained in Mayer’s hematoxylin solution for 5 min and then placed in 0.5% hydrochloric acid in 70% alcohol for 5 s. After the samples were washed twice and dissolved in 1% phosphomolybdic acid aqueous solution, the slides were stained with aniline blue or brilliant green for 5 min. Finally, we dehydrated the specimens in 95% ethyl alcohol several times and added hyalinization with dimethylbenzene. All slides were scanned and digitized using the Digital Pathology Slide Scanner System (Leica Biosystems). The collagen fibers are stained blue, the nuclei are stained black, and the backgrounds are stained red.

### Sample preparation for scRNA-seq

Fresh specimens were minced and enzymatically digested as previously described^60,61^. In brief, the tissues were cut into small pieces and digested in 5% fetal bovine serum (FBS; Gibco, #16000–044) in phosphate-buffered saline (PBS) supplemented with Type VI Collagenase (2 mg/ml, Sigma-Aldrich, #C2139), trypsin inhibitor (1 mg/mL, Sigma-Aldrich, #T6522) and DNase I (1 unit/mL, Millipore Sigma, Germany) for 25–30 min at room temperature with shaking at 100 rpm. Each PDAC tumor sample was digested for two to three cycles depending on tissue texture, and the cells collected from each cycle were merged into one sample. Cell digestion samples were strained through a 70 μm cell strainer and sorted for 4’,6-diamidino-2-phenylindole-negative cells to remove dead cells. Then, we resuspended these sorted cells in PBS containing 3% FBS for the following experiments.

### The 10× library preparation and sequencing

Single-cell suspensions were loaded into a 10× Chromium controller and converted to barcoded scRNA-seq libraries according to the standard protocol of the Chromium Single-Cell 3’ Kit to capture 5000 cells (V2 chemistry). All the remaining steps, including library construction, followed the standard manufacturer’s protocol.

### Public data sources

Public scRNA-seq raw datasets were obtained from Genome Sequence Archive (CRA001160). In brief, samples from two healthy donors and samples from six patients were involved (See Supplementary Table S2)^38^.

### scRNA-seq data processing

All raw read processing was carried out using the Cell Ranger Single-Cell Software Suite (version 3.1.0, 10× Genomics, Inc., CA). In brief, the demultiplexed FASTQ files (150 bp paired-end) were generated using the Cell Ranger *mkfastq* command. The primary data analyses, which included alignment, filtering, barcode counting, and UMI quantification for determining gene transcript counts per cell (generated a gene-barcode matrix), quality control, clustering, and statistical analysis, were performed using the Cell Ranger *count* command. Genes were annotated using Ensembl build 93 and filtered (only genes for protein-coding, long intergenic noncoding RNA, antisense RNA).

### Single-cell gene expression quantification and determination of the major cell types

Raw gene expression matrices generated per sample using Cell Ranger were imported into R (version 3.6.0) and converted to a Seurat object using the Seurat R package (version 3.1.0)^62^. Dead cells and doublets were removed as follows: First, the total number of UMIs and genes and the percentage of UMIs derived from the mitochondrial genome for each cell were counted. Then, the upper bound was calculated as the mean plus two standard deviations (SDs), and the lower bound was calculated as the mean minus two SDs for both the total UMIs and genes. Next, cells with > 15% UMIs derived from the mitochondrial genome were discarded. Finally, cells with total UMIs or genes outside of the upper and lower bounds were removed. For the remaining 55,278 out of 77,121 cells, gene expression matrices were normalized so that the number of UMIs in each cell was equal to 10,000 and log transformed; highly variable genes (HVGs) were selected from the normalized data using the Seurat *SCTransform* function. The top 2000 HVGs were used as features for dimensionality reduction and clustering. The Seurat *RunPCA* functions were performed to select principal components (PCs) that had the most differences to separate the cells. The *RunUMAP* function with parameter “dim = 1:10” was then applied to plot the selected significant PCs. We further performed the batch effect correction using fastMNN^63^ because the batch effect among samples was observed. The Seurat object was split into a list using function SplitObject with parameter “split.by = “Sample”, in which each sample had a sub Seurat object. Then we ordered the sub Seurat object list, in which sample with more cluster number is on the top. Next function RunFastMNN was applied to the list of sub Seurat object with parameter “features = 2500”. The *RunUMAP* function with setting “n.commponents = 6, dim = 1:12” was applied to plot the first 6 “mnn” aligned coordinates. The *FindClusters* function with the “resolution *=* 0.2” parameter was used to cluster cells into different groups. The canonical marker genes were applied to annotate cell clusters as known biological cell types. Cell cycle scores were also calculated using the Seurat *CellCycleScoring* function.

### Reclustering of the cell subtypes

To identify subclusters within cell subtypes, we separately reanalyzed cells that belonged to different cell types. Specifically, we reselected the HVGs for each cell subtype as described above and then applied PC analysis to the selected HVGs for dimensionality reduction. Batch effect correction and UMAP dimensionality reduction using default and graph-based clustering cell reclustering were also performed as described above.

### Identification of marker genes and differentially expressed genes (DEGs)

To identify marker genes for these cell types, we compared the gene expression values of cells from the cluster of interest to that of cells from the rest of the clusters using the Seurat *FindMarkers* function with the default parameter of the “MAST” test. Marker genes were defined based on the following criteria: (1) the average expression value in the cluster of interest was at least 1.2-fold higher than the average expression in the rest of the clusters; (2) Gene expression was detected in > 10% of cells in the cluster of interest; and (3) marker genes should have the highest mean expression in the cluster of interest compared to the rest of the clusters. To identify DEGs for each cell type, we compared the gene expression values of cells between two paired treatments for each cluster of interest using the Seurat *FindMarkers* function with the default parameter of the “MAST” test. Then, criteria applied for marker genes were used as cutoffs to call significant DEGs. Marker gene lists and complete DEG lists without any filtering were used as input for GSEA (V4.03).

### GSEA and GO analysis

GSEA analysis was performed by folding change data from differential expression analysis into GSEA software (Broad Institute). GO analysis was performed with marker genes from each subcluster by tools from DAVID.

### Targeted NGS

Paraffin in formalin-fixed paraffin-embedded (FFPE) sections were removed by xylenes. Genomic DNA was then extracted with QIAamp DNA FFPE Tissue Kit (Qiagen) and quantified by PicoGreen fluorescence assay (Invitrogen). gDNAs were constructed into the libraries with KAPA Hyper Prep Kit (Kapa Biosystems).

For targeted capture, indexed libraries were subjected to probe-based hybridization with a customized NGS panel targeting 733 cancer-related genes, where the probe baits were individually synthesized with 5’-biotinylated 120 bp DNA oligonucleotides (IDT) and repetitive elements were filtered out from intronic baits according to the annotation by UCSC Genome RepeatMasker. The xGen® Hybridization and Wash Kit (IDT) was employed for hybridization enrichment. The captured DNAs were then amplified by PCR, whose final DNA concentrations were determined by Qubit and the DNA sizes were analyzed by Caliper. Libraries were adjusted to 1.05 nM and sequenced in NGS platform illumina Nextseq 500 with Illumina version 4 sequencing kits according to the manufacturer’s instructions. In all, 733 targeted cancer-related genes were listed in Supplementary Table S19.

### Tissue microarrays and immunohistochemistry (IHC)

The tissue microarrays were performed for 94 unselected, primary, and sporadic pancreatic cancer patients treated in Renji Hospital (Pancreatic Cancer Center) and other cooperative hospitals with well-documented clinicopathological information, including patient age, sex, location, tumor differentiation, T stage, lymph node metastasis, distant metastasis, nervous invasion, vascular invasion and follow-up data (ended in March 2016). In total, 94 patients, including 63 males and 31 females, with a median age of 62 years old (ranging from 31 to 78 years old), were enrolled. We obtained follow-up data for 76 patients in this cohort. The overall survival time ranged from 1.75 to 30.00 months, with a median of 14 months.

For the IHC analyses, PDAC tumor tissues and normal tissue were fixed in 10% formalin, embedded in paraffin, and sectioned transversely. Four-micrometer sections were deparaffinized in xylene and rehydrated in graded alcohol. Endogenous peroxidases were blocked by 3% H_2_O_2_, and antigen retrieval was completed after heating in citrate buffer. The primary antibodies used are listed as follows: anti-RGS5 (Abcam, ab196799), anti-PDGFRB (Abcam, ab32570), anti-MYH11 (Abcam, ab133567), anti-periostin (Abcam, ab14041), anti-CRABP2 (Abcam, ab181255), anti-ApoD (Abcam, ab108191), and anti-PLA2G2A (Abcam, ab23705). The tissues were incubated with the primary antibody at 4°C overnight and then incubated with horseradish peroxidase (HRP) (Gene Tech GT Vision III Detection Kit, Shanghai, China) at room temperature for 4 h. Then, PBST was used for washing for ~30 min. The signal was detected with 3,3’-diaminobenzidine solution. All IHC slides were scanned and digitized using the Digital Pathology Slide Scanner System (Leica Biosystems). The staining intensity was quantified as 0 (negative), 1 (weak), or 2 (strong) by two independent reviewers.

Multiplex IF staining was conducted in 9 PDAC samples undergoing scRNA-seq to confirm the result of scRNA. All IF slides were scanned and digitized using Pannoramic MIDI (3DHISTECH Ltd, Hungary). In total, 5–10 high-power fields were taken per patient sample depending on tumor size to quantitate the average number of PLA2G2A^+^, POSTN^+^, and CD8^+^ cells. The cell numbers were quantified by two independent reviewers.

### Cell–cell interactions analysis

Cell–cell interactions based on the expression of known L–R pairs in different cell types were calculated using Cellchat version 0.0.2 (https://github.com/sqjin/CellChat)46. In brief, gene expression data of cells and assigned cell type were used as input for CellChat. First, overexpressed ligands or receptors in one cell group were identified, and then gene expression data were projected onto protein–protein interaction network. The overexpressed L–R interactions were identified if either the ligand or receptor was overexpressed. Next, CellChat was used to infer the biologically significant cell–cell communication by assigning each interaction with a probability value and performing a permutation test. Finally, communication networks were visualized using circle plot and signaling pathways visualized using bubble plot.

### TCGA and online database

In our provisional data set, 178 patients from TCGA database were included (TCGA, Firehose Legacy).

### Pilot study

The study is an open-label, single-arm, single-center, non-randomized pilot study. This study was conducted in RenJi Hospital and approved by the Ethics Committee of Renji Hospital (IRB approval number: KY[2019]035). Expression of PLA2G2A in the stroma was detected as an explored biomarker.

PD-1 antibody SHR-1210 used in our study is a humanized monoclonal antibody from Jiangsu Hengrui Medicine Co.,Ltd., and the heavy chain is immunoglobulin G4 (IgG4), the light chain is immunoglobulin κ.

### Statistical analysis

SPSS 16.0 software (Chicago, IL, USA) was used for statistical analyses. The association between CAF-related marker gene expression and clinicopathological characteristics was statistically determined using the Pearson *χ*^2^ test. Overall survival (death from any cause) was analyzed using the Kaplan–Meier method, and the results were compared using the log-rank test. Two-sided Mann–Whitney *U* test was used unless mentioned, and *P* values < 0.05 were considered statistically significant. Univariate and multivariate Cox regression analyses were performed in the R statistical environment to assess the effects of prognostic factors.

## Supplementary information

Fig. S1

Fig. S2

Fig. S3

Fig. S4

Fig. S5

Fig. S6

Fig. S7

Fig. S8

Fig. S9

Fig. S10

Fig. S11

Fig. S12

Fig. S13

Table S1

Table S2

Table S3

Table S4

Table S5

Table S6

Table S7

Table S8

Table S9

Table S10

Table S11

Table S12

Table S13

Table S14

Table S15

Table S16

Table S17

Table S18

Table S19

## Data Availability

All scRNA-seq data reported in this paper are available in the CNGB Sequence Archive (CNSA) of China National GeneBank DataBase (CNGBdb) with accession number CNP0001768.
