# Supplementary material for: Single-cell analysis of pancreatic ductal adenocarcinoma identifies a novel fibroblast subtype associated with poor prognosis but better immunotherapy response
Source: Cell Discov. 2021 May 25;7:36. doi: 10.1038/s41421-021-00271-4 (PMC8149399; doi:10.1038/s41421-021-00271-4)
Supplement: Supplementary file 1 — Fig. S1 [file 41421_2021_271_MOESM1_ESM.pdf]

Supplementary Figure S1.

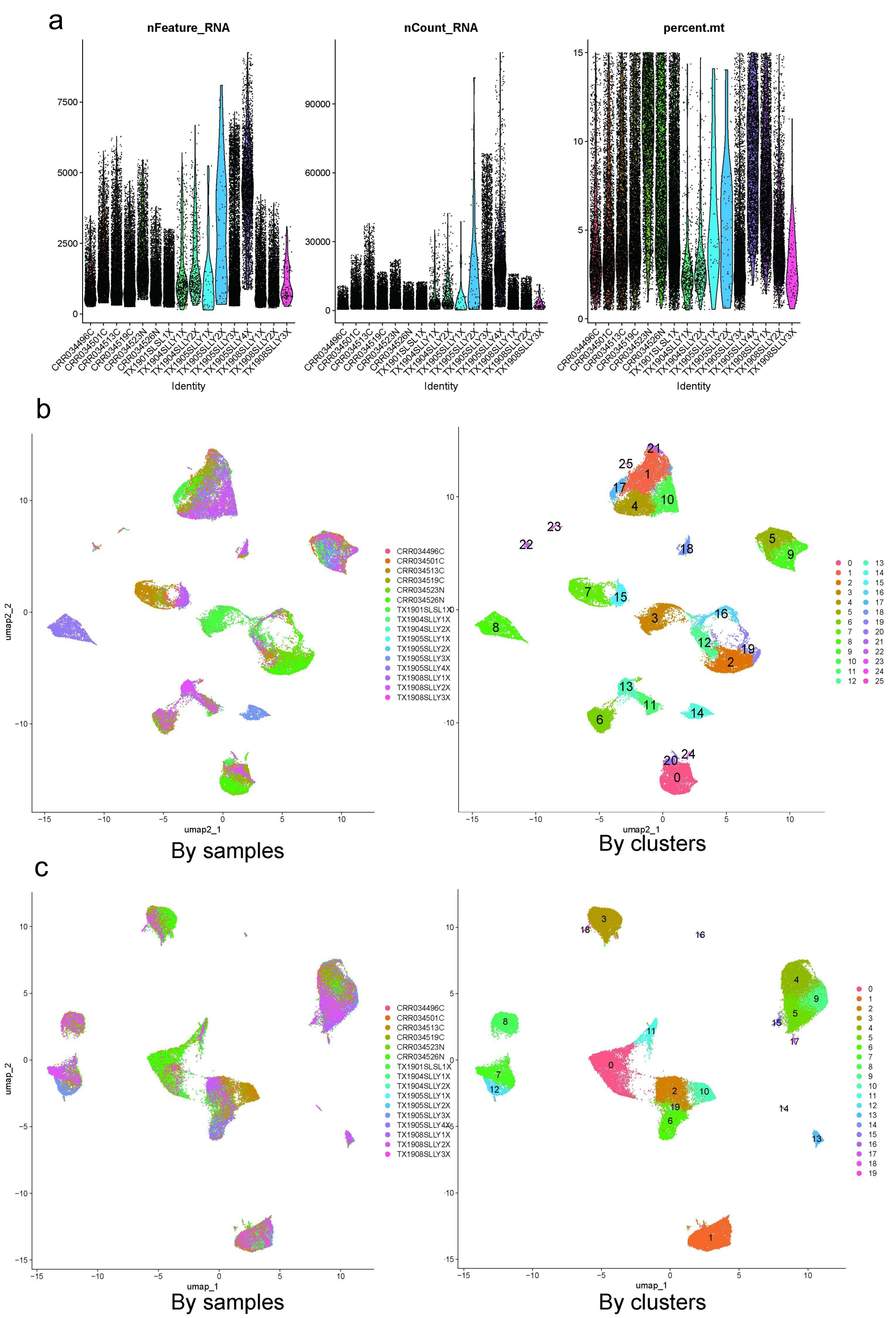

Supplementary Figure S1.

a，Application of MNN-based batch correction to scRNA-seq data, showing the data quality and the condition before filtering; **b**，Cell population of human PDAC single cell transcriptomes without Batch effect correction, each color-coded region indicates one sample or one cluster; **c**，Cell population of human PDAC single cell transcriptomes with batch effect correction.
