# Supplementary material for: Single-cell analysis of pancreatic ductal adenocarcinoma identifies a novel fibroblast subtype associated with poor prognosis but better immunotherapy response
Source: Cell Discov. 2021 May 25;7:36. doi: 10.1038/s41421-021-00271-4 (PMC8149399; doi:10.1038/s41421-021-00271-4)
Supplement: Supplementary file 2 — Fig. S2 [file 41421_2021_271_MOESM2_ESM.pdf]

Supplementary Figure S2.

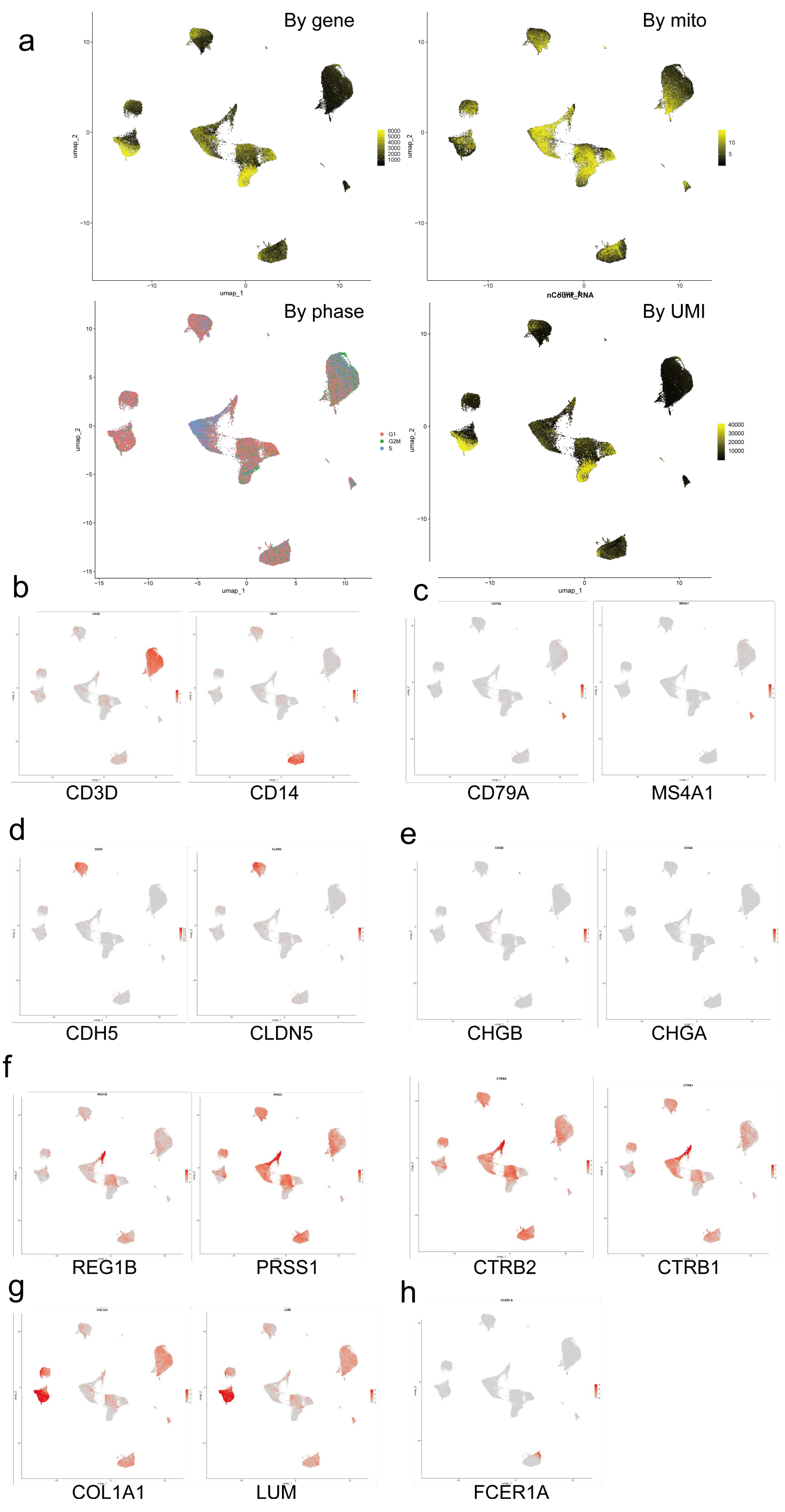

Supplementary Figure S2.

a，Different cell transcript state of the clusters(by gene, mito, phase and UMI); b-h，Feature plots show known marker genes at single cell resolution of several important clusters; **b** represents T cell and monocyte/macrophage; **c** represents B or plasma cell; **d** represents endothelial cell; **e** represents endocrine cell; **f** represents acinar cell; **g** and **h** represent fibroblasts and dendritic Cell.
