# Supplementary material for: Single-cell analysis of pancreatic ductal adenocarcinoma identifies a novel fibroblast subtype associated with poor prognosis but better immunotherapy response
Source: Cell Discov. 2021 May 25;7:36. doi: 10.1038/s41421-021-00271-4 (PMC8149399; doi:10.1038/s41421-021-00271-4)
Supplement: Supplementary file 3 — Fig. S3 [file 41421_2021_271_MOESM3_ESM.pdf]

Supplementary Figure S3.

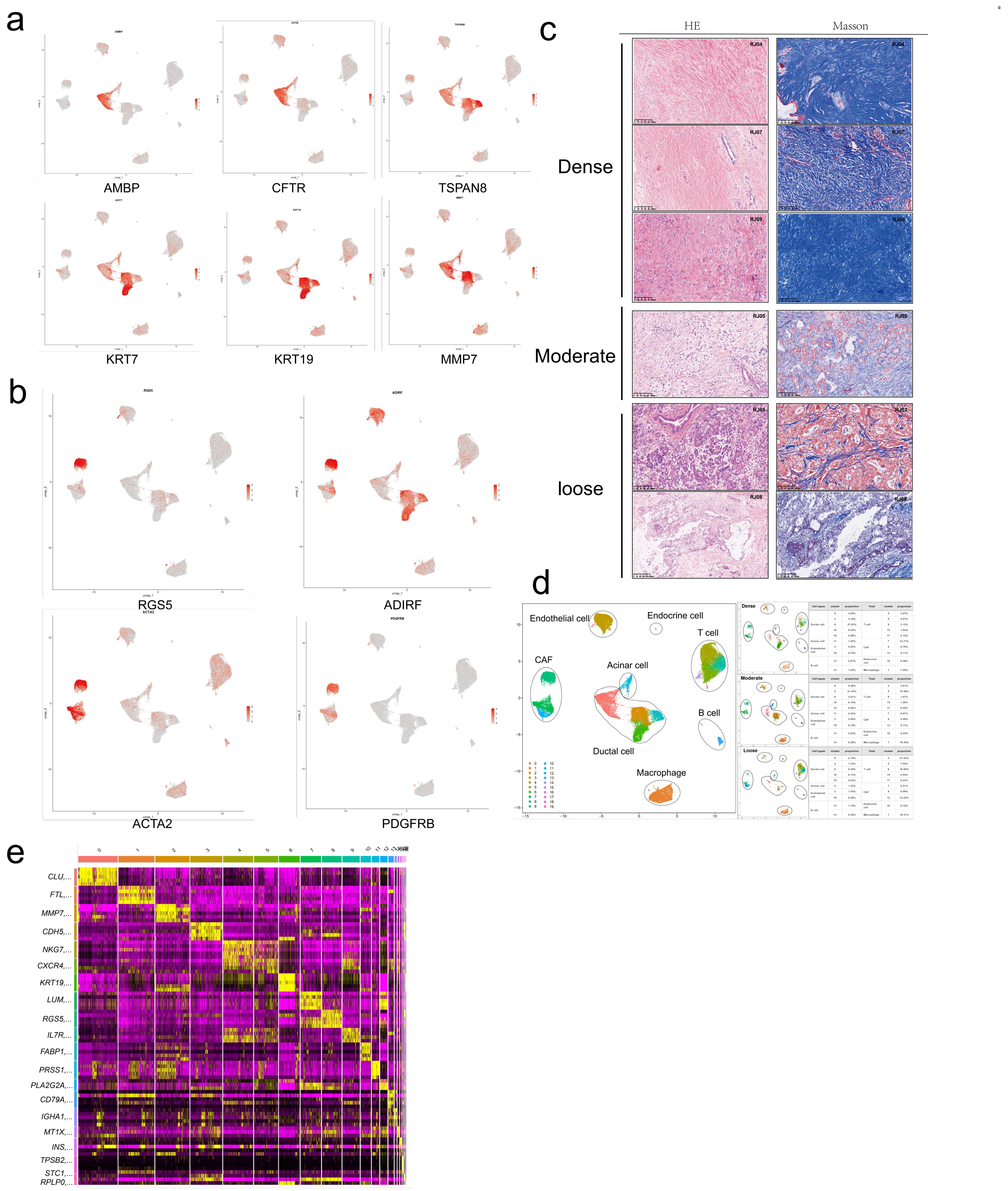

Supplementary Figure S3.
a, Feature plots show the marker genes of ductal cell; **b,** Feature plots show the marker genes of stellate cell. **c,** HE and Masson staining of representative PDAC tissues from both dense-, moderate- and loose-type of PDACs, Scale bar, 100 μm. **d,** Unsupervised clustering of viable cells was represented as UMAP. 19 cluster and 8 cell types were identified. The cell proportion in different degree of desmoplasia (dense, moderate and loose) was showed in the tables. **e,** Single-cell profiling heatmap of marker genes among all 20 clusters.
