# Supplementary material for: Single-cell analysis of pancreatic ductal adenocarcinoma identifies a novel fibroblast subtype associated with poor prognosis but better immunotherapy response
Source: Cell Discov. 2021 May 25;7:36. doi: 10.1038/s41421-021-00271-4 (PMC8149399; doi:10.1038/s41421-021-00271-4)
Supplement: Supplementary file 4 — Fig. S4 [file 41421_2021_271_MOESM4_ESM.pdf]

Supplementary Figure S4.

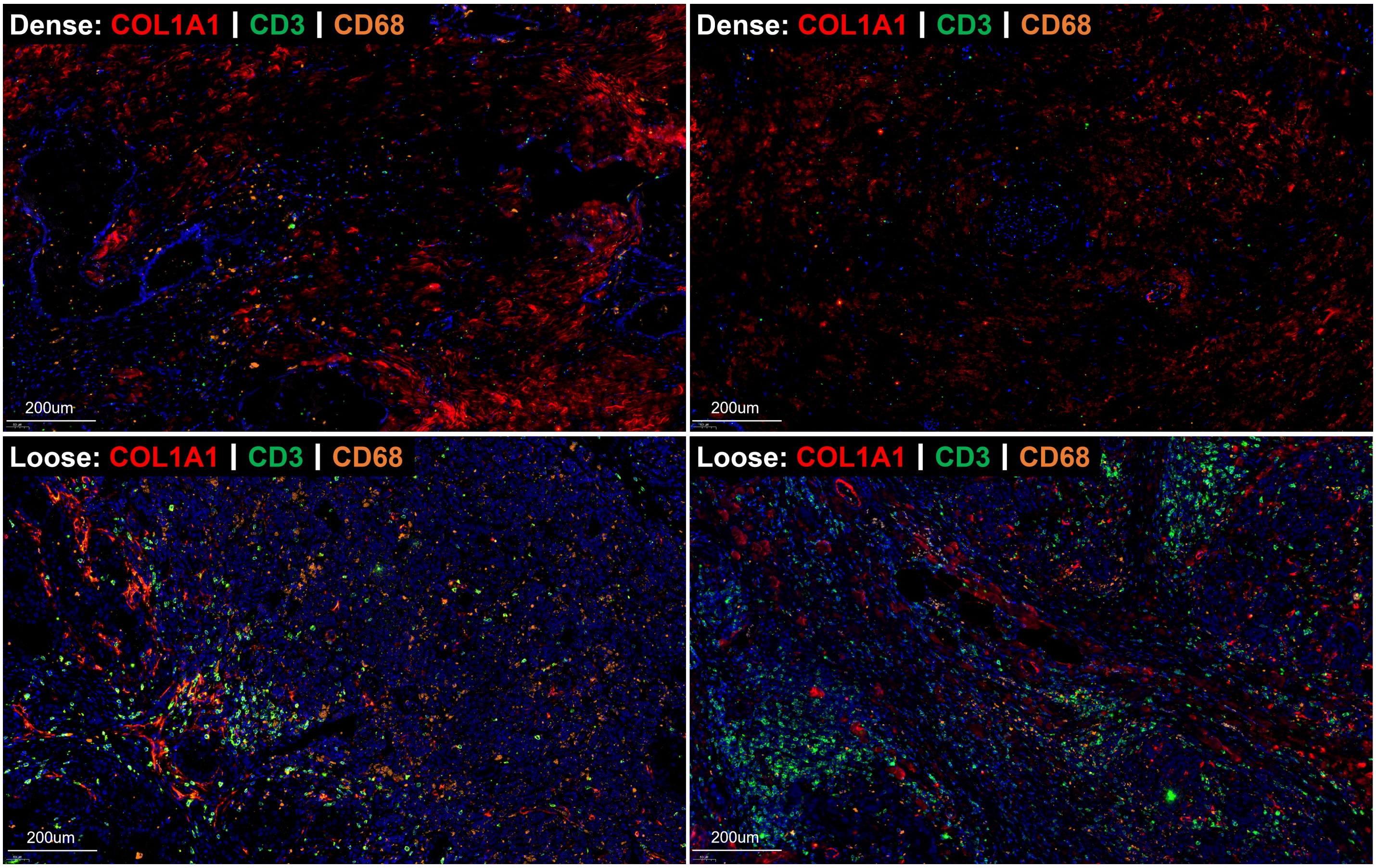

Supplementary Figure S4.
Multiplex immunofluorescence staining was conducted in nine PDAC samples undergoing scRNA-seq to confirm the changes in T cell and macrophage in dense- and loose-type PDACs. Representative images (two dense-type PDACs and two loose type PDACs from 9 PDAC samples, including one dense case and one loose case already shown in Fig 1e) were shown. The result shown that loose stroma was associated with the increase of infiltrating T cells and macrophages. COL1A1 for CAF (red), CD3 for T cell (green), CD68 for macrophage (orange).
