# Supplementary material for: Single-cell analysis of pancreatic ductal adenocarcinoma identifies a novel fibroblast subtype associated with poor prognosis but better immunotherapy response
Source: Cell Discov. 2021 May 25;7:36. doi: 10.1038/s41421-021-00271-4 (PMC8149399; doi:10.1038/s41421-021-00271-4)
Supplement: Supplementary file 5 — Fig. S5 [file 41421_2021_271_MOESM5_ESM.pdf]

Supplementary Figure S5.

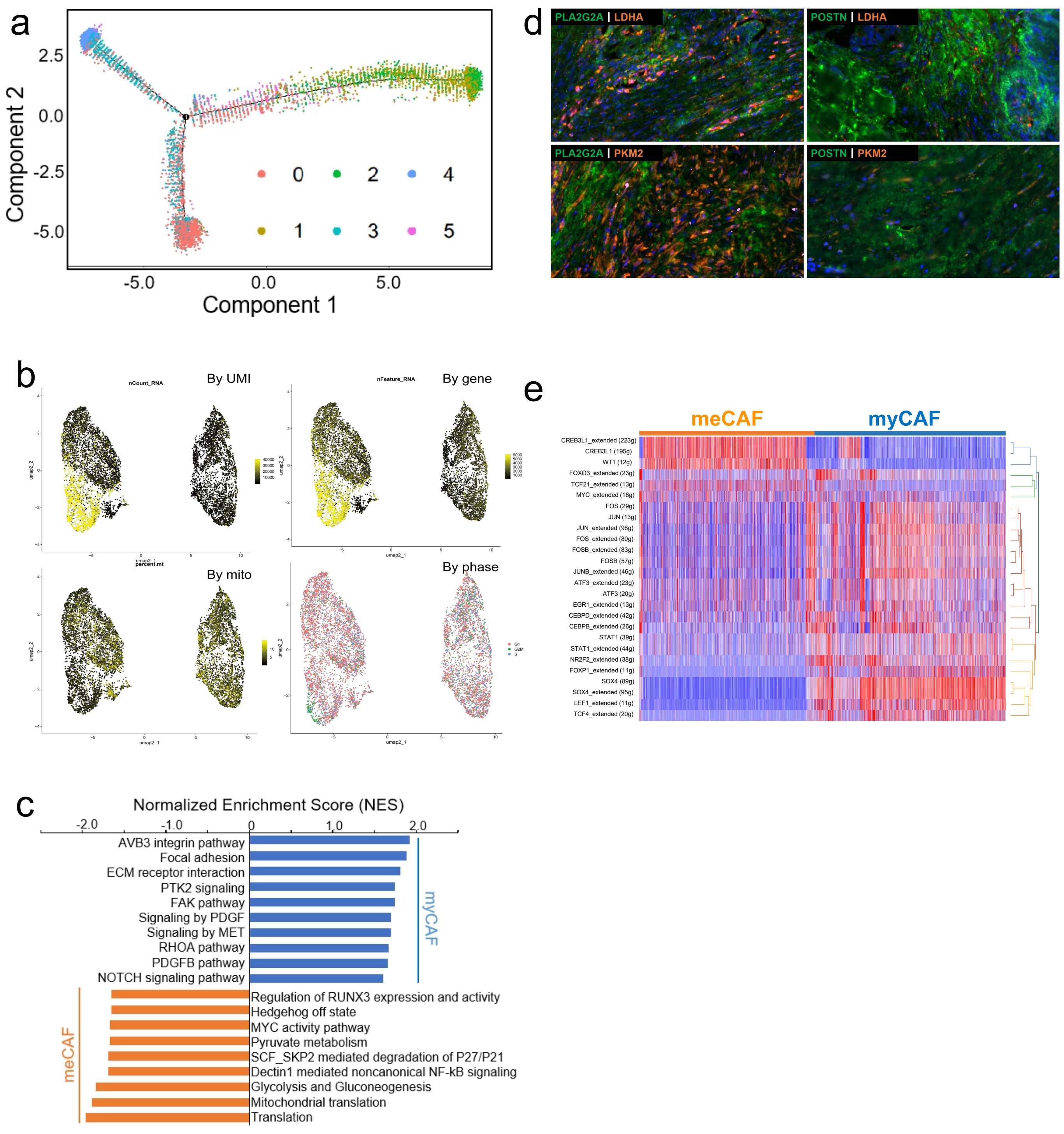

Supplementary Figure S5.
a, Pseudo-time of CAF in all PDAC samples inferred by Monocle2. Monocle2 pseudotime trajectory of C1 and C2 differentiation into C0, C3, C4 and C5. Cells are colored by cluster. Each point corresponds to a single cell. **b,** Different cell transcript state of CAF (by gene, mito, phase and UMI). **c,** GSEA of enriched pathways in myCAFs (subcluster C3) or meCAFs (subcluster C4) based on differential expression. **d,** Multiplex staining of CAF markers, LDHA and PKM2. Compared to myCAF (POSTN+ CAFs), the meCAFs (PLA2G2A+ CAFs) had more co-staining with LDHA and PKM2. **e,** SCENIC TF analysis revealing the distinct upstream transcription factors between myCAFs and meCAFs.
