# Supplementary material for: Single-cell analysis of pancreatic ductal adenocarcinoma identifies a novel fibroblast subtype associated with poor prognosis but better immunotherapy response
Source: Cell Discov. 2021 May 25;7:36. doi: 10.1038/s41421-021-00271-4 (PMC8149399; doi:10.1038/s41421-021-00271-4)
Supplement: Supplementary file 6 — Fig. S6 [file 41421_2021_271_MOESM6_ESM.pdf]

Supplementary Figure S6.

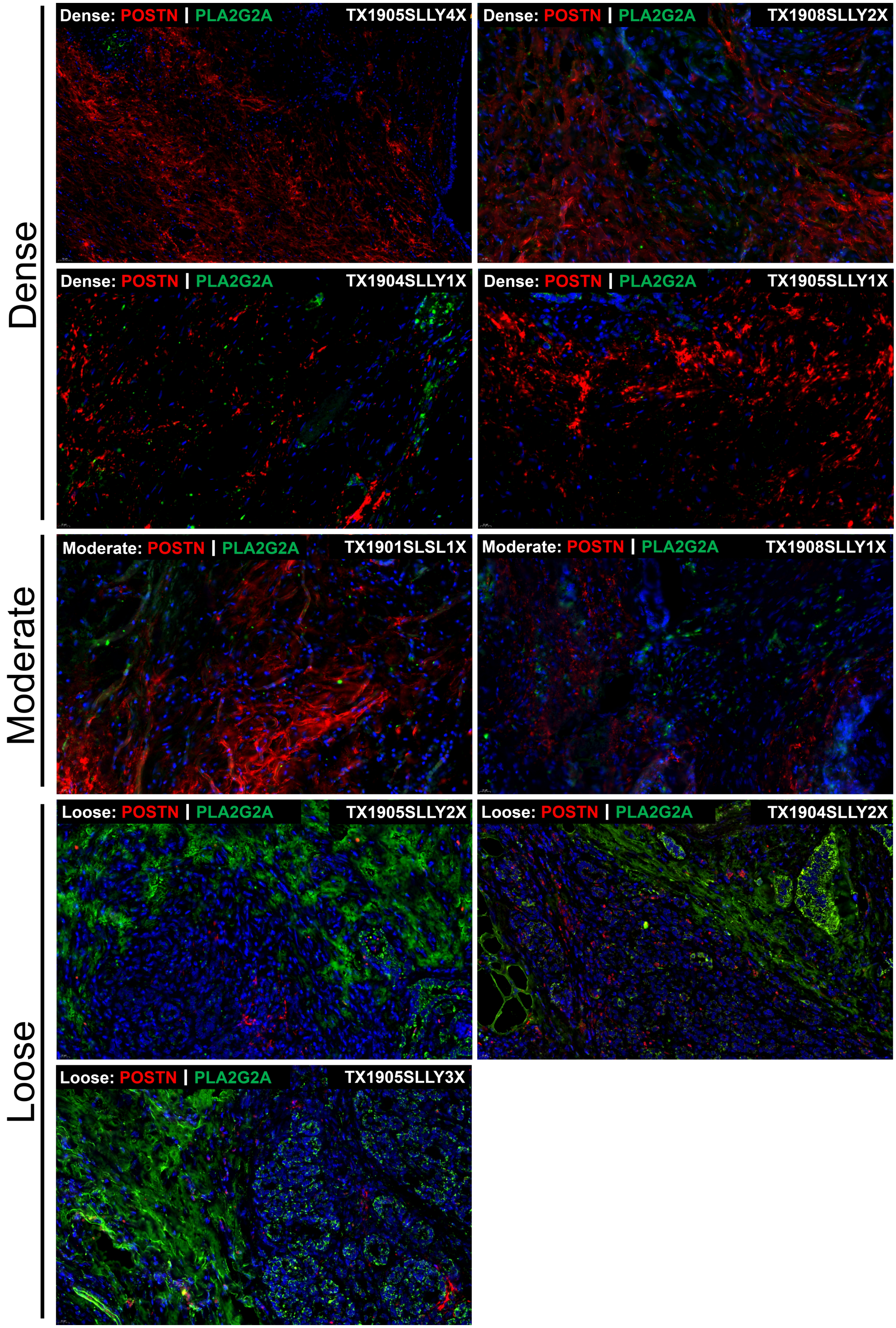

Supplementary Figure S6. Expression of myCAF marker (POSTN, red) and meCAF marker (PLA2G2A, green) in 9 PDAC samples undergoing scRNA-seq. The results showed that high expression of myCAF marker in dense-type PDAC while meCAF marker was highly expressed in loose-type PDAC.
