# Supplementary material for: Single-cell analysis of pancreatic ductal adenocarcinoma identifies a novel fibroblast subtype associated with poor prognosis but better immunotherapy response
Source: Cell Discov. 2021 May 25;7:36. doi: 10.1038/s41421-021-00271-4 (PMC8149399; doi:10.1038/s41421-021-00271-4)
Supplement: Supplementary file 7 — Fig. S7 [file 41421_2021_271_MOESM7_ESM.pdf]

Supplementary Figure S7.

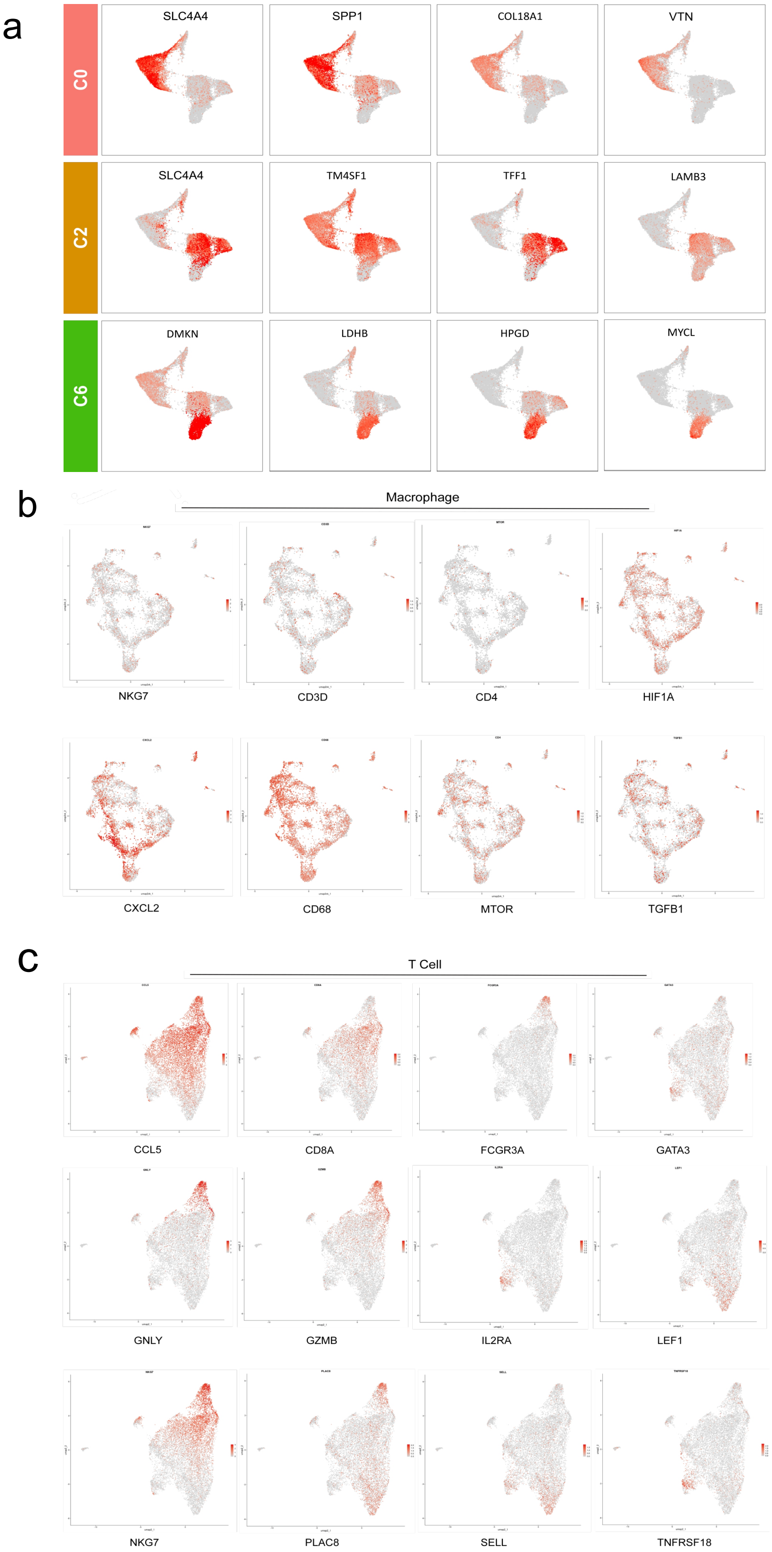

Supplementary Figure S7.
a, Feature plots show the expression level of representative markers across three ductal cell subclusters (C0, C2, C6). **b-c,** Feature plots show the expression levels of representative markers for macrophage and T cell. Color key from grey to red indicates relative expression levels from low to high.
