# Supplementary material for: Single-cell analysis of pancreatic ductal adenocarcinoma identifies a novel fibroblast subtype associated with poor prognosis but better immunotherapy response
Source: Cell Discov. 2021 May 25;7:36. doi: 10.1038/s41421-021-00271-4 (PMC8149399; doi:10.1038/s41421-021-00271-4)
Supplement: Supplementary file 8 — Fig. S8 [file 41421_2021_271_MOESM8_ESM.pdf]

Supplementary Figure S8.

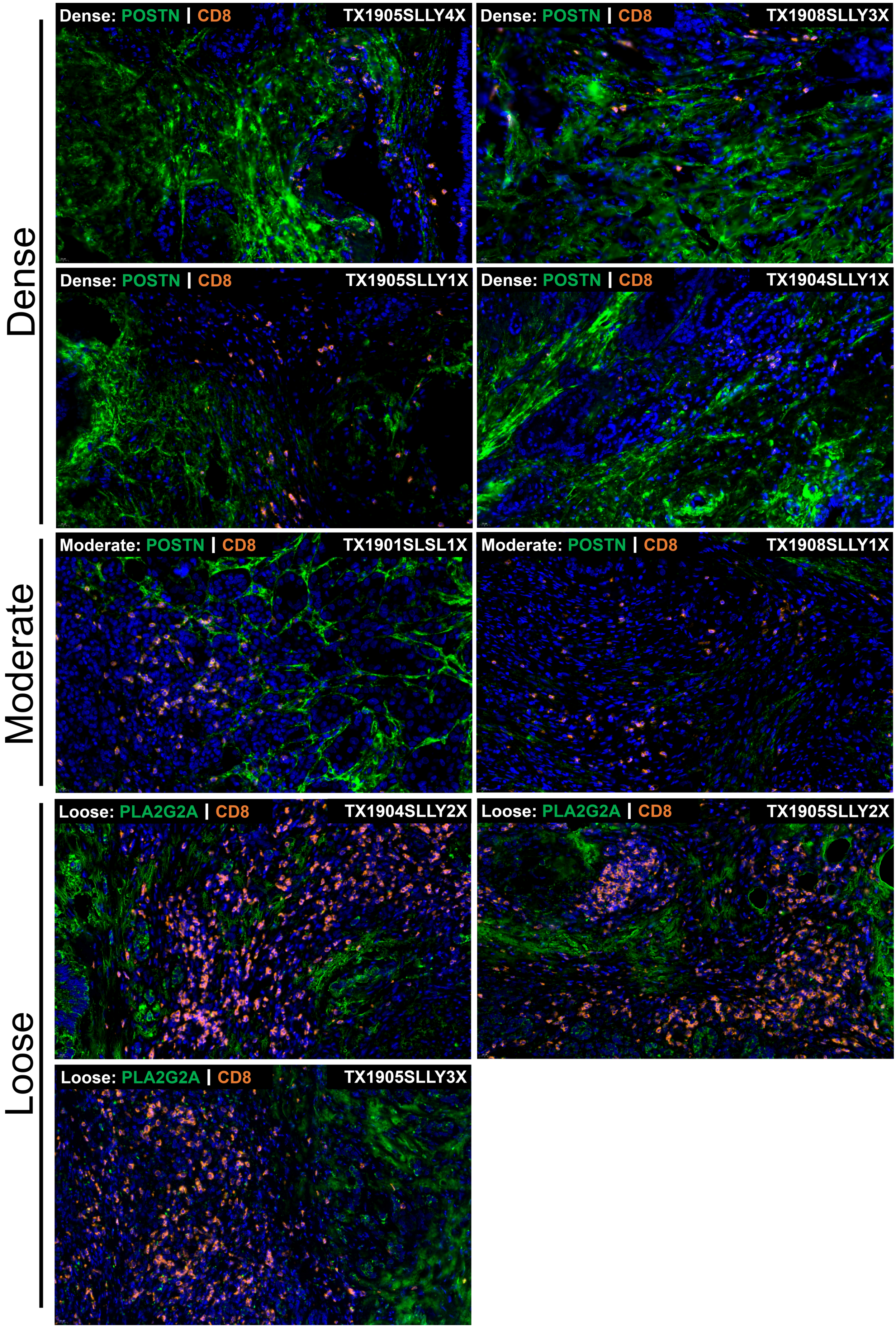

Supplementary Figure S8. Expression of CD8 in 9 PDAC samples undergoing scRNA-seq. The result showed much more infiltrating CD8+ T cells in loose-type PDAC than in dense-type PDAC.
