# Supplementary material for: Single-cell analysis of pancreatic ductal adenocarcinoma identifies a novel fibroblast subtype associated with poor prognosis but better immunotherapy response
Source: Cell Discov. 2021 May 25;7:36. doi: 10.1038/s41421-021-00271-4 (PMC8149399; doi:10.1038/s41421-021-00271-4)
Supplement: Supplementary file 9 — Fig. S9 [file 41421_2021_271_MOESM9_ESM.pdf]

Supplementary Figure S9.

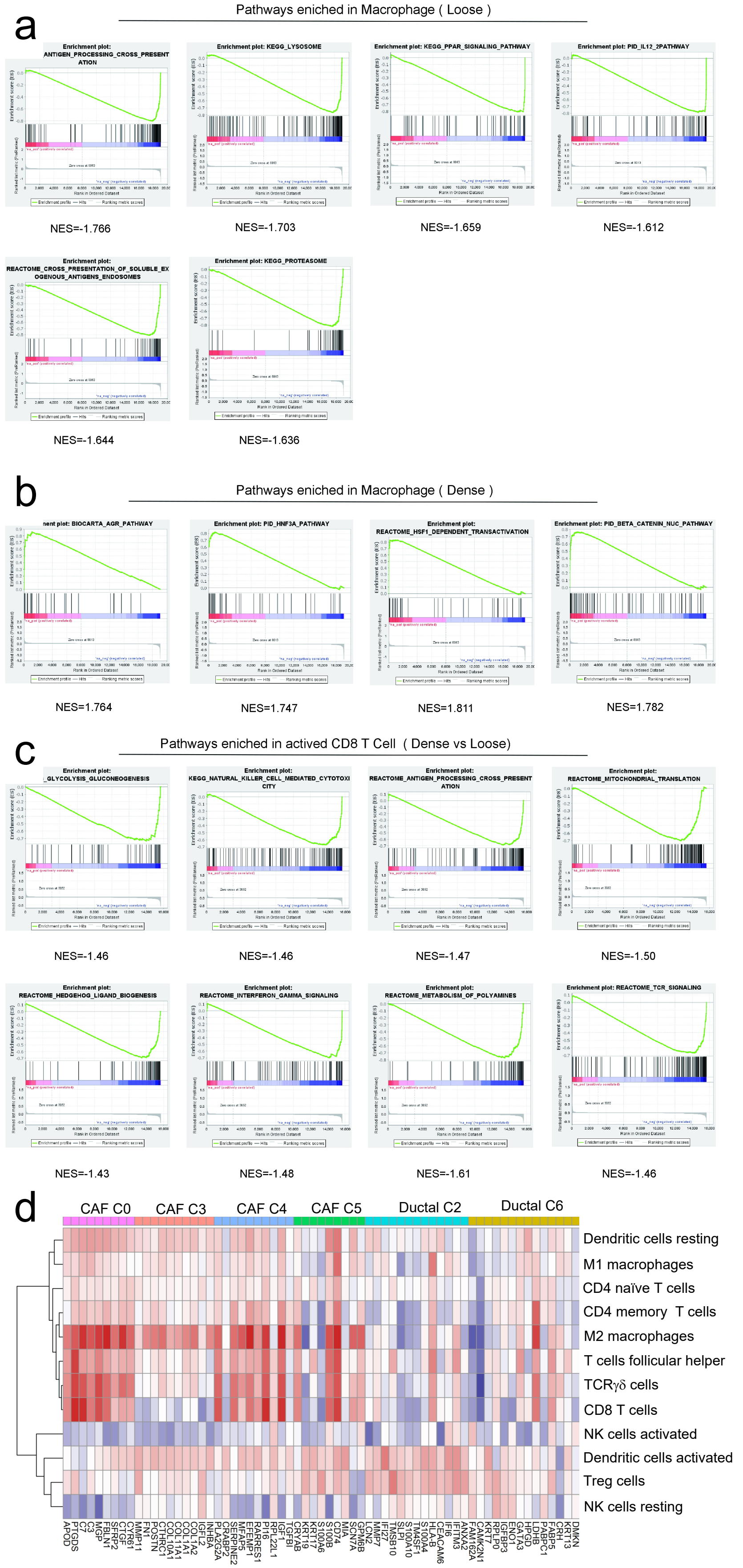

Supplementary Figure S9.

a-b, GSEA analysis of enriched pathways in macrophage comparing loose-type against dense-type of PDAC. **c,** GSEA analysis of enriched pathways in actived CD8+ T cell in loose-type PDAC. **d,** Correlation analysis of marker gene expression in ductal cell types and CAF subclusters with immune cell populations in TCGA database.
