# Supplementary material for: Single-cell analysis of pancreatic ductal adenocarcinoma identifies a novel fibroblast subtype associated with poor prognosis but better immunotherapy response
Source: Cell Discov. 2021 May 25;7:36. doi: 10.1038/s41421-021-00271-4 (PMC8149399; doi:10.1038/s41421-021-00271-4)
Supplement: Supplementary file 10 — Fig. S10 [file 41421_2021_271_MOESM10_ESM.pdf]

Supplementary Figure S10.

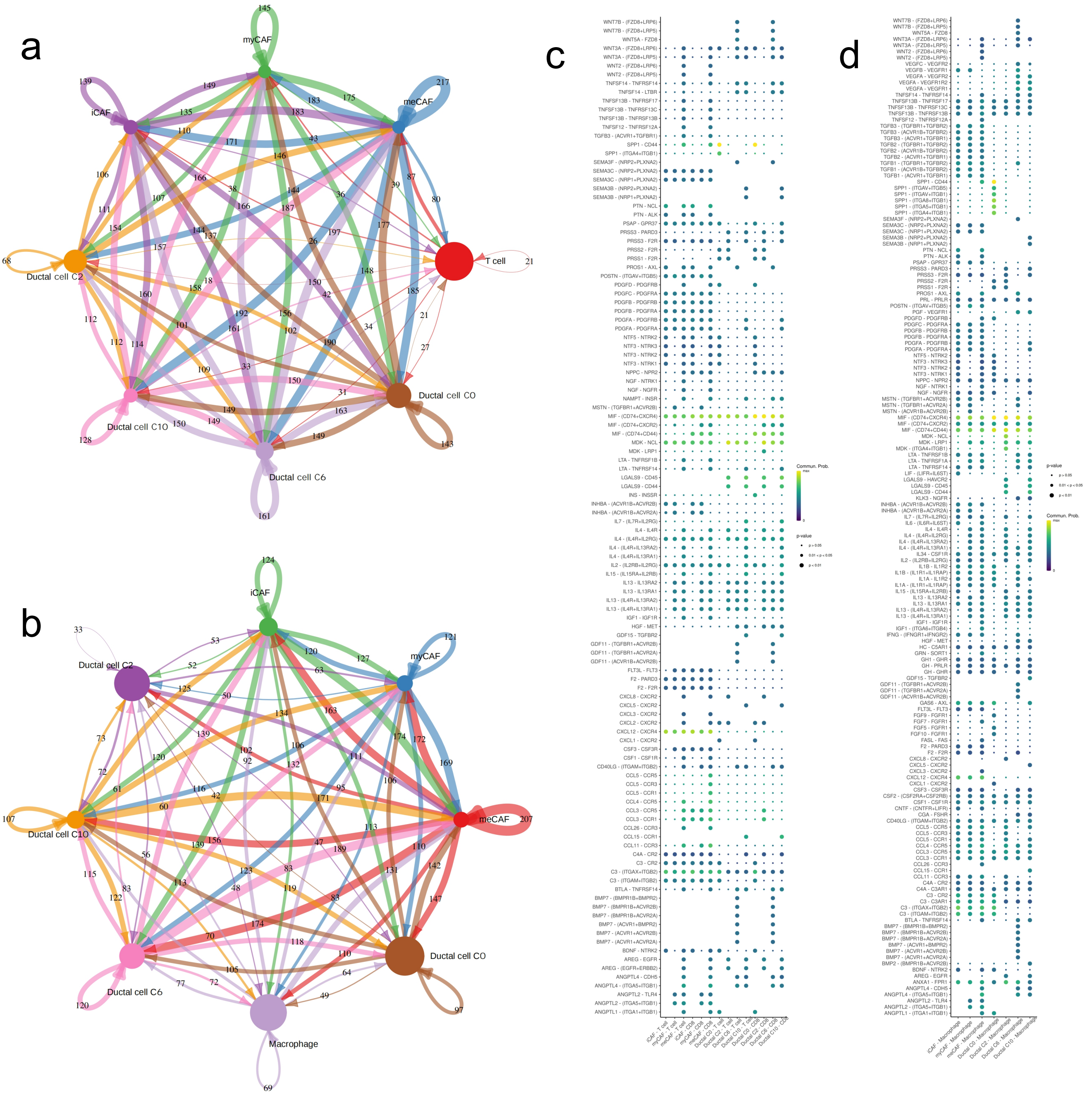

Supplementary Figure S10.

Cell chat analysis for ductal cells, CAFs and immune cells. a Number of significant ligand-receptor pairs between any two pairs of cell populations (myCAF, meCAF, iCAF, Ductal C0, Ductal C2, Ductal C6,Ductal C10 and T cell). b Number of significant ligand-receptor pairs between any two pairs of cell populations (myCAF, meCAF, iCAF, Ductal C0, Ductal C2, Ductal C6, Ductal C10 and macrophage). c Significant ligand-receptor pairs sending signals from CAF subclusters and ductal subclusters to T cells. d Significant ligand-receptor pairs sending signals from CAF subclusters and ductal subclusters to macrophages.
