# Supplementary material for: Single-cell analysis of pancreatic ductal adenocarcinoma identifies a novel fibroblast subtype associated with poor prognosis but better immunotherapy response
Source: Cell Discov. 2021 May 25;7:36. doi: 10.1038/s41421-021-00271-4 (PMC8149399; doi:10.1038/s41421-021-00271-4)
Supplement: Supplementary file 11 — Fig. S11 [file 41421_2021_271_MOESM11_ESM.pdf]

Supplementary Figure S11.

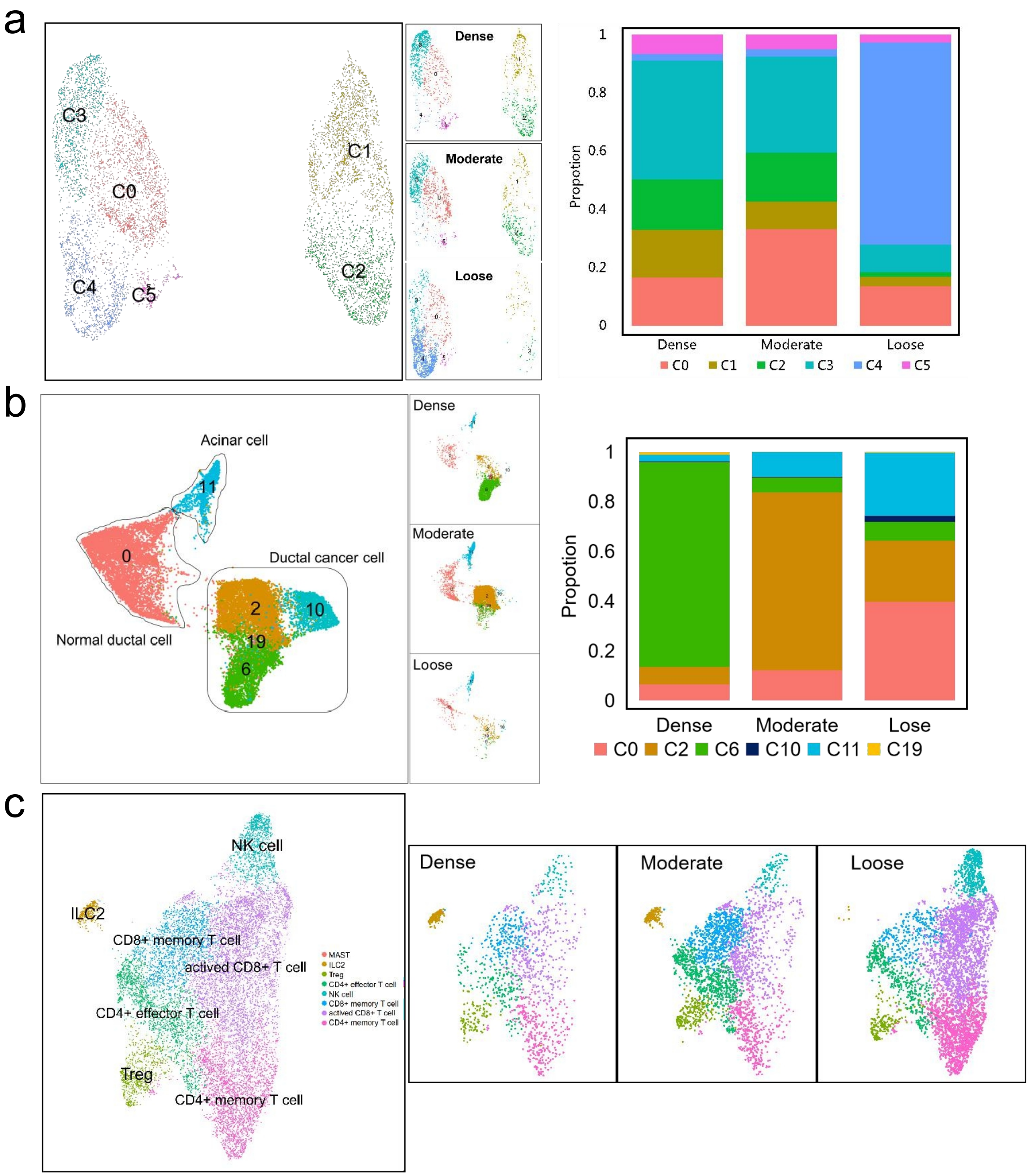

Supplementary Figure S11.

a, UMAP of acinar cell and ductal cell in dense, moderate- and loose-type. Proportions of each CAF subclusters in dense, moderate and loose stroma are shown. C3 (myCAF) is the major component of CAF in dense and moderate stroma, while C4 (meCAF) is the major CAF in loose stroma. **b,** UMAP of acinar cell and ductal cell in dense-, moderate- and loose-type. Different proportion of 6 subclusters in dense, loose and moderate stroma. **c,** T cells，NK cell, CD8+ memory T cell, CD8+ actived T cell, CD4+memory T cell, CD4+ effector T cell, ILC2 and Treg cell were identified. The distributions of each cell types in dense, loose and moderate stroma are shown.
