# Supplementary material for: Single-cell analysis of pancreatic ductal adenocarcinoma identifies a novel fibroblast subtype associated with poor prognosis but better immunotherapy response
Source: Cell Discov. 2021 May 25;7:36. doi: 10.1038/s41421-021-00271-4 (PMC8149399; doi:10.1038/s41421-021-00271-4)
Supplement: Supplementary file 12 — Fig. S12 [file 41421_2021_271_MOESM12_ESM.pdf]

Supplementary Figure S12.

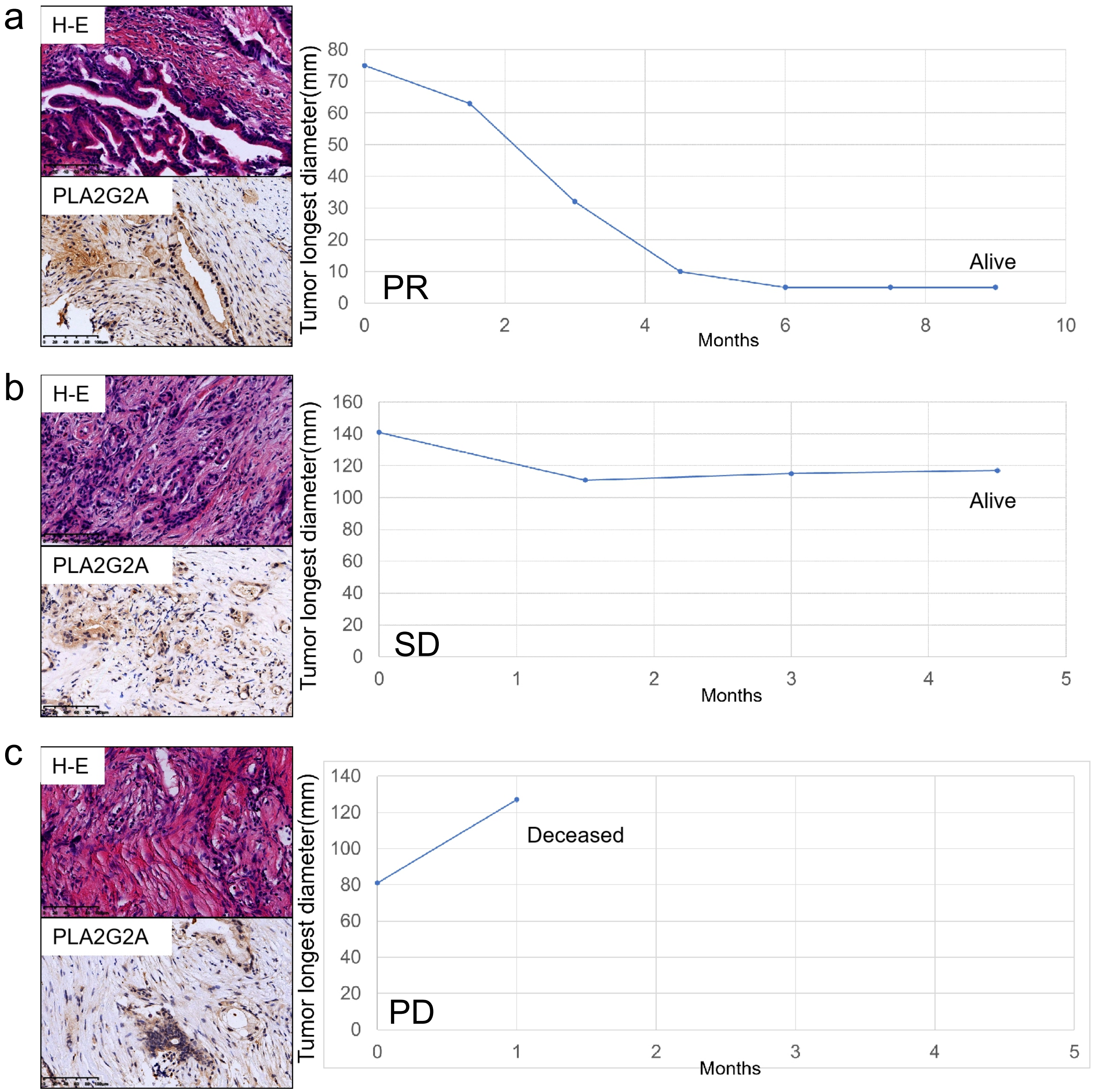

Supplementary Figure S12.
a-c, These curves showed the changes in the tumor longest diameter of PDAC patients with different response (PR, SD and PD, respectively). Scale bar, 100 μm.
