# Supplementary material for: Single-cell analysis of pancreatic ductal adenocarcinoma identifies a novel fibroblast subtype associated with poor prognosis but better immunotherapy response
Source: Cell Discov. 2021 May 25;7:36. doi: 10.1038/s41421-021-00271-4 (PMC8149399; doi:10.1038/s41421-021-00271-4)
Supplement: Supplementary file 13 — Fig. S13 [file 41421_2021_271_MOESM13_ESM.pdf]

Supplementary Figure S13.

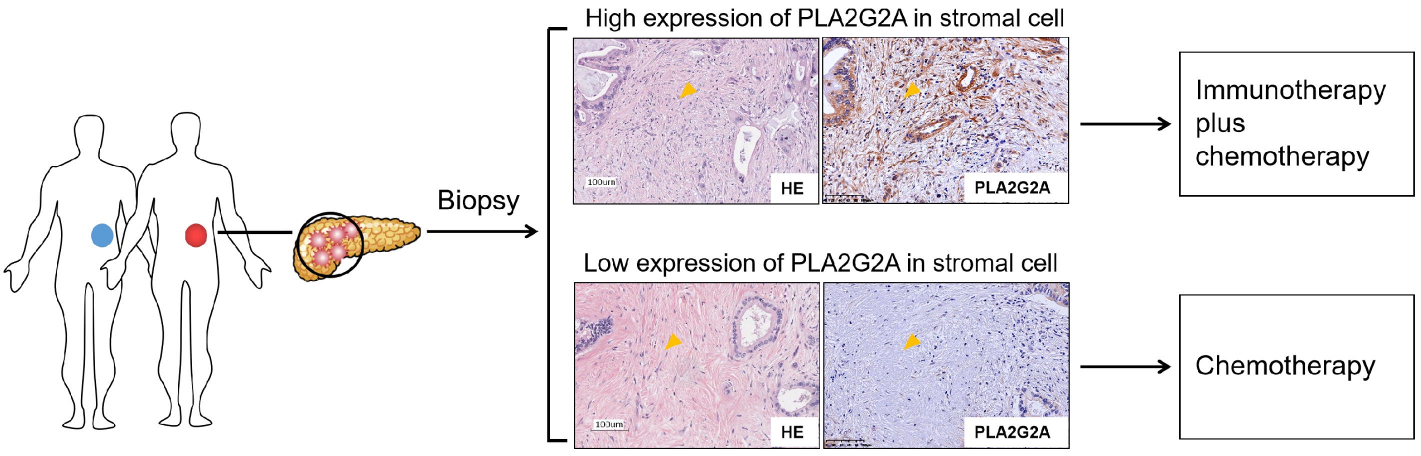

Supplementary Figure S13.
PDAC patients received biopsy before treatment, and then HE staining and immunohistochemical staining of PLA2G2A expression were performed in paraffin sections. The expression of PLA2G2A in stromal cells was used to predict the response to immunotherapy, so as to select optimal treatment for them.
